# A Study of the Effect of 5 at.% Sn on the Micro-Structure and Isothermal Oxidation at 800 and 1200 °C of Nb-24Ti-18Si Based Alloys with Al and/or Cr Additions

**DOI:** 10.3390/ma13010245

**Published:** 2020-01-06

**Authors:** Zhen Xu, Claire Utton, Panos Tsakiropoulos

**Affiliations:** Department of Materials Science and Engineering, Sir Robert Hadfield Building, The University of Sheffield, Mappin Street, Sheffield S1 3JD, UK; zhen_xu@outlook.com (Z.X.); c.utton@sheffield.ac.uk (C.U.)

**Keywords:** niobium silicide based alloys, solidification, oxidation, silicides, Nb_3_Sn, Nb_5_Sn_2_Si, solid solution, C14-NbCr_2_ Laves phase, tin effect

## Abstract

This paper presents the results of a systematic study of Nb-24Ti-18Si based alloys with 5 at.% Sn addition. Three alloys of nominal compositions (at.%), namely Nb-24Ti-18Si-5Cr-5Sn (ZX4), Nb-24Ti-18Si-5Al-5Sn (ZX6), and Nb-24Ti-18Si-5Al-5Cr-5Sn (ZX8), were studied to understand how the increased Sn concentration improved oxidation resistance. In all three alloys there was macrosegregation, which was most severe in ZX8 and the primary βNb_5_Si_3_ transformed completely to αNb_5_Si_3_ after heat treatment. The Nb_ss_ was not stable in ZX6, the Nb_3_Sn was stable in all three alloys, and the Nb_ss_ and C14-NbCr_2_ Laves phase were stable in ZX4 and ZX8. The 5 at.% Sn addition suppressed pest oxidation at 800 °C but not scale spallation at 1200 °C. At both temperatures, a Sn-rich area with Nb_3_Sn, Nb_5_Sn_2_Si, and NbSn_2_ compounds developed below the scale. This area was thicker and continuous after oxidation at 1200 °C and was contaminated by oxygen at both temperatures. The contamination of the Nb_ss_ by oxygen was most severe in the bulk of all three alloys. Nb-rich, Ti-rich and Nb and Si-rich oxides formed in the scales. The adhesion of the latter on ZX6 at 1200 °C was better, compared with the alloys ZX4 and ZX8. At both temperatures, the improved oxidation was accompanied by a decrease and increase respectively of the alloy parameters VEC (Valence Electron Concentration) and δ, in agreement with the alloy design methodology NICE (Niobium Intermetallic Composite Elaboration). Comparison with similar alloys with 2 at.% Sn addition showed (a) that a higher Sn concentration is essential for the suppression of pest oxidation of Nb-24Ti-18Si based alloys with Cr and no Al additions, but not for alloys where Al and Cr are in synergy with Sn, (b) that the stability of Nb_3_Sn in the alloy is “assured” with 5 at.% Sn addition, which improves oxidation with/out the presence of the Laves phase and (c) that the synergy of Sn with Al presents the “best” oxidation behaviour with improved scale adhesion at high temperature.

## 1. Introduction

Structural metallic materials for applications at high temperatures must have a balance of mechanical properties and oxidation resistance. Coated and internally cooled Ni-based superalloys are used in gas turbine engines close to their high temperature limit, which is imposed by the melting temperature of Ni. The search for new alloys with similar or better capabilities at higher temperatures has concentrated on Nb-silicide based alloys that have lower densities, significantly higher solidus temperatures in excess of 1900 °C and can offer notable mechanical properties [1]. For example, some of these alloys can have compressive yield strength of about 1800 MPa at room temperature, 1200 MPa at 1000 °C and 500 MPa at 1200 °C [2]. The oxidation resistance of Nb-silicide based alloys was improved dramatically when the key alloying elements Al, Cr, Si, and Ti were in synergy with other transition and refractory metals, for example Hf, Mo, and simple metal and metalloid elements, e.g., Sn [1,3,4,5,6]. 

In the case of Sn, the early research [3,4] concentrated on alloys where its concentration was low to avoid the formation of the A15-Nb_3_Sn compound. In 2007, Geng et al [5] reported that the addition of Sn in the Nb-24Ti-18Si-5Al-5Cr-5Hf-5Sn-2Mo alloy (i) suppressed pest oxidation at 800 °C and (ii) improved the adhesion of the scale that formed at 1200 °C, which did not separate from the substrate, and linked the improved oxidation with Sn enrichment of the substrate below the scale where at 1200 °C the Nb_3_Sn and Nb_5_Sn_2_Si intermetallics were observed. The latter compound can be in equilibrium with Nb_3_Sn and Nb_5_Si_3_ at least up to 1200 °C in the Nb-Si-Sn system [7], has the same crystal structure as βNb_5_Si_3_ (prototype W_5_Si_3_) as does the Nb_5_Sn_2_Al that can be in equilibrium with the A15-Nb_3_Al compound [8]. Formation of Sn rich areas below the scales that formed at 800 °C on the alloys Nb-23Ti-5Si-5Al-5Hf-5V-2Cr-2Sn and Nb-30Ti-10Si-5Cr-5Sn-3Fe-2Al-2Hf (nominal compositions) was reported by our group in 2008 [9]. A later study by Knittel et al [10] considered the effect of Sn for a wider range of Sn concentrations in Nb-25Ti-16Si-8Hf-2Cr-2Al-*x*Sn (*x* = 0,2,4,5,6,8) alloys (nominal compositions) and confirmed the elimination of pesting at 815 °C, and the presence of NbSn_2_ and/or Sn at this temperature and Nb_5_Sn_2_Si with M_5_Si_3_ (M = transition/refractory metal) below the scales that formed at 1100 and 1200 °C. Cheng et al. [11] studied the orientation relationship between Nb_ss_ and αNb_5_Si_3_ in the Nb-20Ti-18Si-4Hf-5Cr-3Al-1.5Sn alloy (nominal composition) but did not report on its oxidation behaviour.

Many questions remain unanswered about how Sn improves the oxidation of Nb-silicide based alloys. For example: (a) How low or high should the concentration of Sn be in the alloy? (b) Is the stability of Nb_3_Sn in the microstructure an essential requirement for oxidation resistance? (c) Is the presence of other elements that form A15 compounds (e.g., Al (Nb_3_Al), Mo (Mo_3_Sn)), Si (Nb_3_Si), V (V_3_Sn)) or TM_5_Sn_2_X (TM = Nb,Ti, X = Si,Al) compounds critical for oxidation resistance? (d) Why the adherence of scales is poor at high temperatures in alloys with Sn? (e) Could the synergy of Sn with other alloying additions improve the adherence of the scales?

In all the aforementioned studies [5,6,7,8,9,10], the role played by Sn in low and high temperature oxidation was masked owing to the presence of elements that are known to also improve oxidation resistance when in synergy with Al, Cr, Si, and Ti (e.g., Hf in [10] or Hf and Mo in [12]). For example, Hf is predicted to segregate to the surface, like Sn. Furthermore, in [10], the addition of Hf at a rather high concentration, which is typical of the MASC alloy, stabilized the hexagonal Nb_5_Si_3_ that is undesirable for creep [1]. The motivation for the research presented in this paper was to make new contributions in the physical metallurgy of Nb-silicide based alloys that would help us answer some of the above questions. Recently, a systematic study which aspired to find out how Al and Cr individually or simultaneously, in synergy with 2 at.% Sn, improved oxidation of Nb-24Ti-18Si silicide based alloys, reported that the Nb_3_Sn compound was stable even at this low Sn concentration and confirmed the formation of Sn rich areas below the scale where Nb_5_Sn_2_Si was formed together with other Sn rich intermetallics, such as NbSn_2_ and Nb_3_Sn [13]. In this paper, the systematic study is expanded to find out how a higher concentration of Sn in basically the same alloys (see next section) would affect their microstructures and isothermal oxidation at 800 and 1200 °C.

The structure of the paper is as follows. First, the microstructures of the cast and heat treated alloys are discussed, followed by the results for their oxidation at 800 °C and 1200 °C separately. The discussion first considers the macrosegregation in the cast alloys, then their solidification and the stability of phases in their microstructures, followed by the discussion of oxidation kinetics and microstructures at each oxidation temperature. 

## 2. Why 2 and 5 at.% Sn?

The design methodology NICE [14] utilizes relationships between the parameters δ, Δχ, and VEC that describe the alloying behaviour of (1) Nb-silicide based alloys and (2) the phases that can be present in their microstructures [15,16,17,18,19]. NICE makes use of the relationships between the aforementioned parameters and (i) the concentrations of solutes in (a) alloy and (b) phases and (ii) the creep rate at different temperatures and stresses, (iv) the weight change in isothermal oxidation at 800 and 1200 °C, and (v) the Si macrosegregation to design alloy compositions, predict properties, and assist the selection of alloys for alloy development research. 

The starting point(s) in NICE is (are) property goal(s), not a specific microstructure. The motivation for the research presented in this paper was discussed in the previous section. Our prime interest was to advance the current understanding of how Sn improves the oxidation of Nb-silicide based alloys. However, Nb-silicide based alloys must also have adequate creep, for which additions of Hf and refractory metals are essential [1,14] but unfortunately can mask the so-called “Sn-effect” (see previous section). Thus, our focus in [13] and in this paper was on Nb-silicide based alloys of the Nb-Ti-Si-Al-Cr-Sn system. 

For this research, the property goals were zero weight change at 800 and 1200 °C (the “ideal” case) and creep rate at 1200 °C, and 100 MPa better than that of the single crystal Ni-based superalloy CMSX-4, which has been ascertained experimentally and is 2 × 10^−6^ s^−1^. The aforementioned parameters were calculated and the concentrations of each of the above solute additions in Nb were derived as described in NICE [14]. The calculations gave the alloy compositions (at.%) Nb-24.4Ti-18.3Si-5.25Al-4.95Cr-5.3Sn and Nb-22.8Ti-18.5Si-4.75Al-4.3Cr-2.5Sn for which the predicted creep rates respectively were 3.3 × 10^−7^ s^−1^ and 1.05 × 10^−7^ s^−1^, lower than that of CMSX-4. NICE also indicated that with increasing Sn concentration, (a) the creep rate and (b) the macrosegregation of Si will increase. Based on these results the nominal compositions of the alloys of this study were selected (see next section) as well as those of the low Sn content alloys that were studied in [13].

## 3. Experimental

The alloys ZX4, ZX6 and ZX8 of nominal compositions (at.%), respectively Nb-24Ti-18Si-5Cr-5Sn, Nb-24Ti-18Si-5Al-5Sn, and Nb-24Ti-18Si-5Al-5Cr-5Sn were prepared in the form of 20 g buttons using arc melting with a water cooled copper crucible in a Ti gettered Argon atmosphere and high purity (better than 99.99 wt.%) elements. In this paper, we refer to these alloys as the higher Sn content alloys, compared with the low Sn content alloys that were reported in [13], namely the alloys Nb-24Ti-18Si-5Cr-2Sn (ZX3), Nb-24Ti-18Si-5Al-2Sn (ZX7), and Nb-24Ti-18Si-5Al-5Cr-2Sn (ZX8). Specimens for heat treatments were wrapped in Ta foil and heat treated under a constant flow of Ti gettered Argon at 1500 °C (ZX4 and ZX6) or 1450 °C (ZX8) for 100 h [5,9,12]. A NETZSCH STA 49 F3 Jupiter thermal analyser (NETZSCH GmbH, Selb, Germany) supported by the NETZSCH Proteus software was used for the isothermal oxidation experiments at 800 and 1200 °C for which cubic (3 × 3 × 3 mm^3^) specimens were cut and ground to 1200 grit. Samples were heated at 3 °C/min from room temperature to 800 or 1200 °C. An isothermal hold for 100 h at temperature was performed under a flow of air (20 mL/min).

The as cast, heat treated, and oxidised alloys were characterised using X ray diffraction (XRD) and scanning electron microscopy (SEM) equipped with energy dispersive spectrometers (EDS) and an electron probe micro analyser (EPMA) equipped with a wavelength dispersive spectrometer (WDS). Specimens were prepared as discussed in [13]. For the XRD experiments, a Siemens 5000 X-ray diffractometer (HiltonBrooks Ltd., Crew, UK) with monochromatic Cu-K_α_ radiation was used and the specimens were scanned using 0.02° step and two theta (2θ) from 20 to 100 degrees. For the identification of phases, the JCPDS (Joint Committee of Powered Diffraction Standard) data was used. An Inspect F SEM (ThermoFisher Scientific, Hillsboro, OR, USA) was used to study the microstructures in back scattered electron (BSE) mode. Microanalyses were performed in a Joel JSM 6400 SEM (JEOL Ltd., Tokyo, Japan) equipped with an Oxford instruments INCA system (Oxon, UK) for quantitative EDS with elemental standards and a Cameca SX100 EPMA (Cameca, Gennevilliers, France) with spatial resolution of 1 μm and equipped with WDS. The instrument calibration was carried out by analysing reference materials with known compositions. The reference materials and their composition used in this work are listed in the Supplementary Table S1. At least 10 analyses were taken from each large area and phase, which are referred to as large area analysis and spot analysis, respectively. The maximum, minimum, and average values and the standard deviation are given in the tables that present the microanalysis data. X-ray maps were taken using a Philips XF30 FEG SEM fitted with a Bruker Quantax analyser (Bruker AXS Ltd., Coventry, UK) and ESPRIT software. The software included data for N and O and supported the identification of nitrides and oxides in the alloys. 

## 4. Results

### 4.1. Cast Alloys

The actual compositions of the as cast alloys are given in Table 1. These were the average values of all the large area analyses taken from the bottom, bulk, and top of the as cast buttons. There was macrosegregation in all three alloys, namely of Cr, Si, and Ti in ZX4, of Si and Ti in ZX6, and of Al, Cr, Si, and Ti in ZX8, as shown in Table 2. In the latter, the macrosegregation of an element i (MACi) is given as the difference between the maximum and minimum analysis values, i.e., as C_max_^i^–C_min_^i^ [20]. The macrosegregation of Si (MACSi) was most severe in ZX4 and ZX8, and of Ti in ZX6. The alloy ZX8 was the most heavily macrosegregated. 

**Figure 1:**
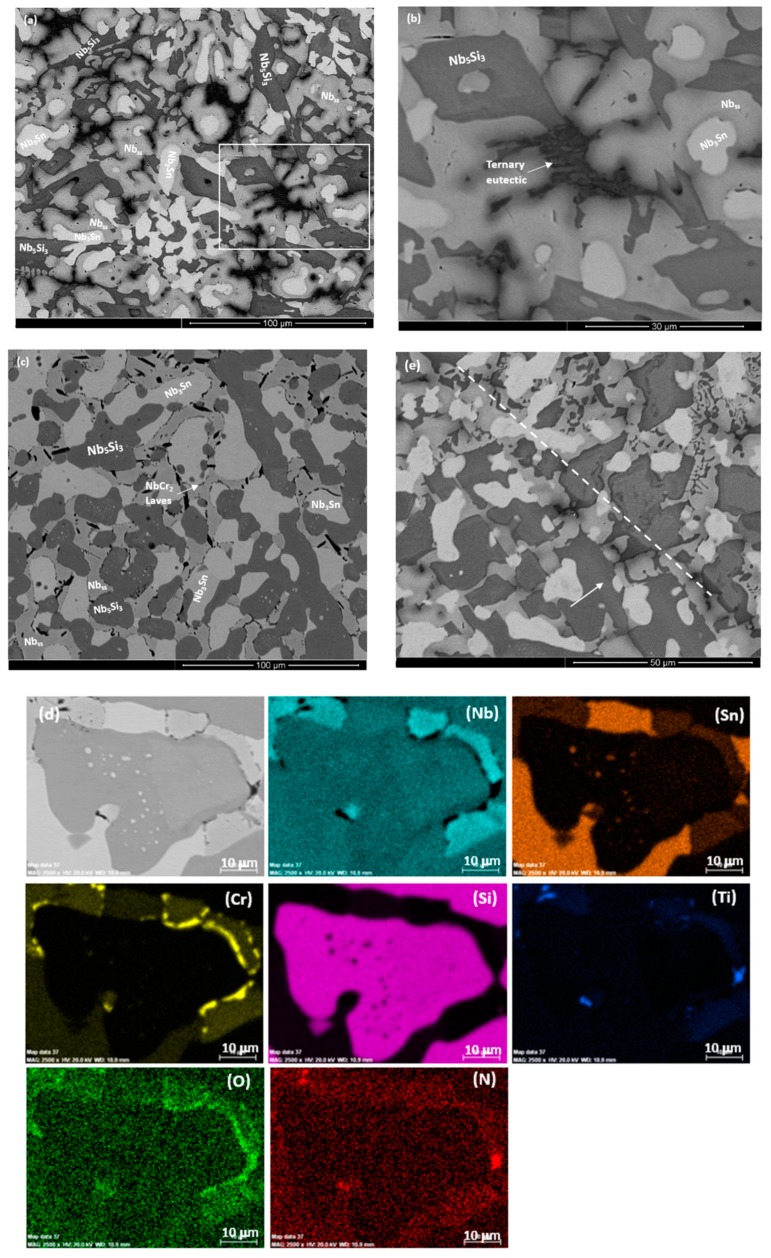
(**a**–**c**,**e**) BSE images of the microstructure (**a**,**e**) in the bottom of the as cast and (**c**) the heat-treated alloy ZX4. (**b**) shows details of area indicated by rectangle in (**a**). (**d**) X-ray maps showing the NbCr_2_ Laves phase and the Sn rich precipitates formed in the αNb_5_Si_3_. In (**e**) the arrow points away from the water cooled crucible wall and the dashed line indicates the change in microstructure from the bottom to the bulk (see text).

**Figure 2:**
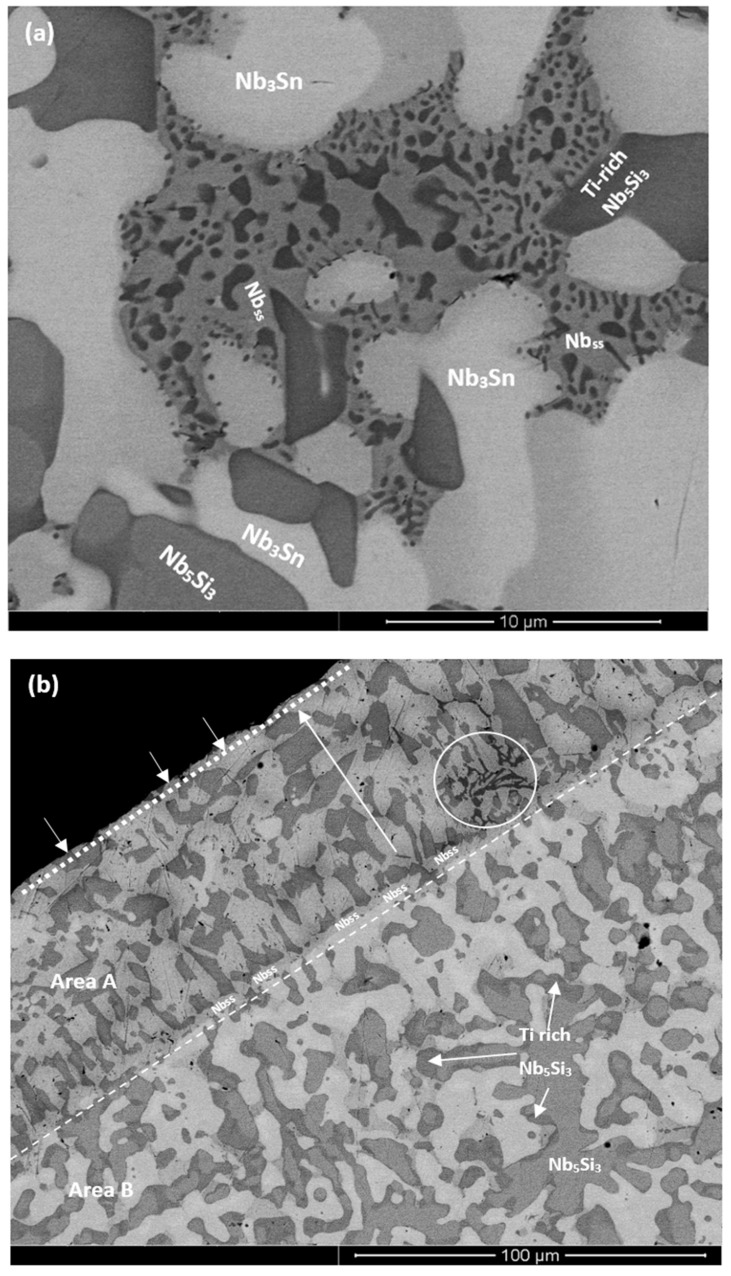

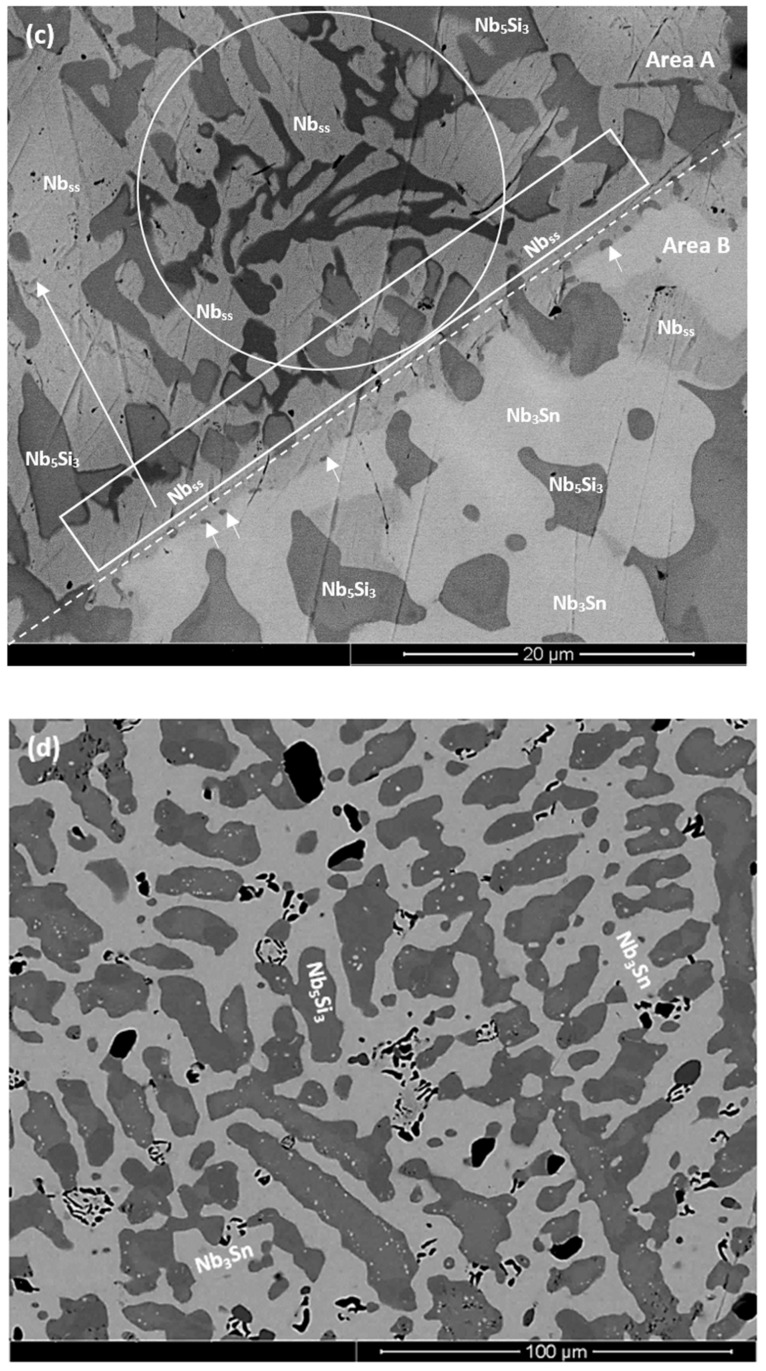

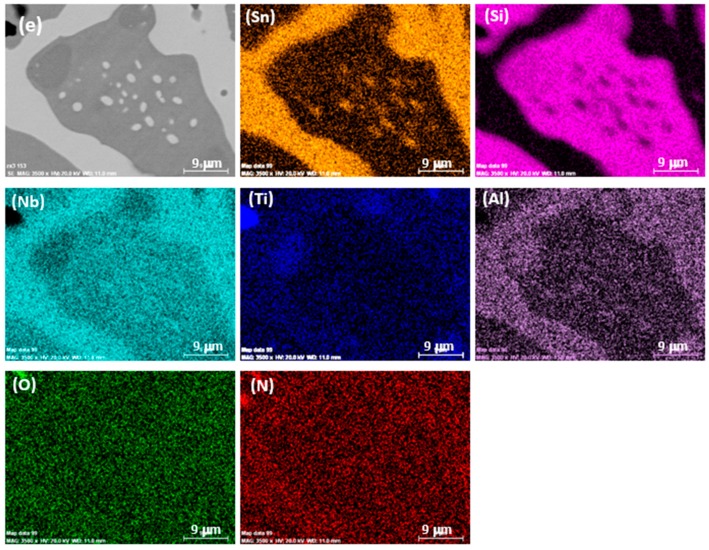
BSE images of the microstructure of the as cast (**a**–**c**) and heat treated (**d**) alloy ZX6 and (**e**) X-ray maps of the heat-treated alloy. (**a**) top, and (**b**,**c**) bottom of the button. (**b**,**c**) show microstructure formed next to the water-cooled crucible. For areas A and B see text. Dashed line shows Nb_ss_ interface between areas A and B. Dotted line with short arrows in (**b**) indicates part of the button in contact with the crucible wall. The arrow in area A shows direction of heat dissipation. The circle shows area where Ti rich Nb_5_Si_3_ formed in area A. For short arrows in (**c**), see text.

**Figure 3:**
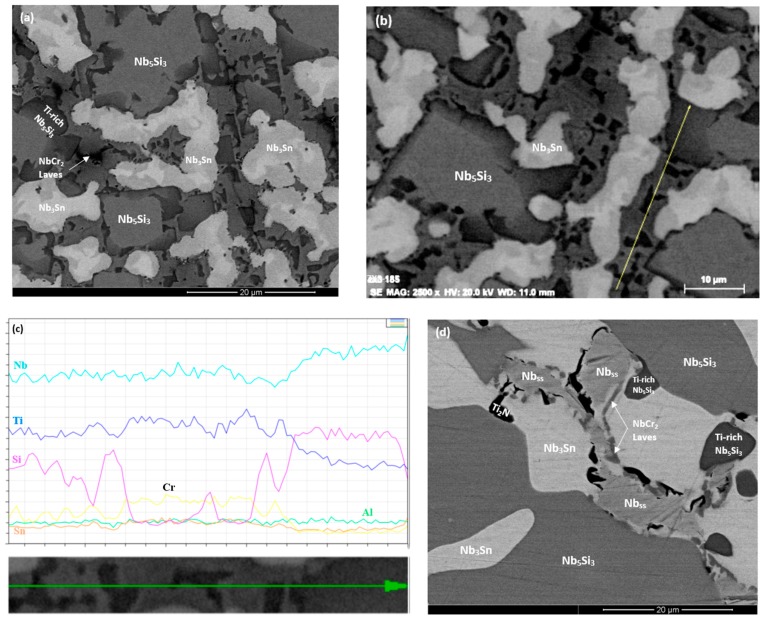
(**a**,**b**,**d**) BSE images of the microstructure (**a**), (**b**) of the as cast and (**d**) heat treated alloy ZX8. (**a**) top, and (**b**,**d**) bulk of button. (**c**) line-scan along the line shown in (**b**).

**Figure 4:**
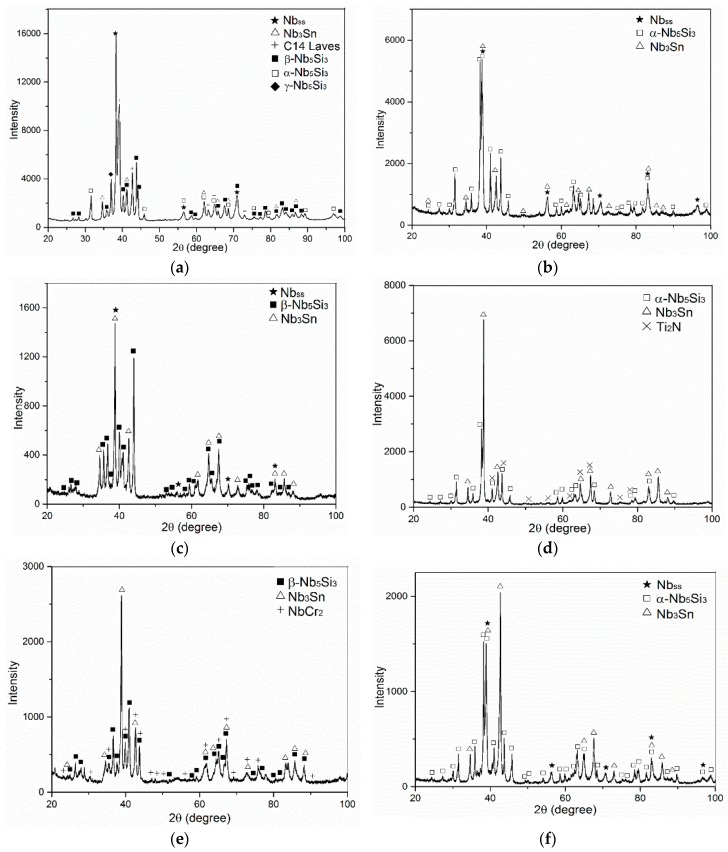
X-ray diffractograms of the as cast alloys (**a**) ZX4, (**c**) ZX6, (**e**) ZX8 and the heat treated alloys (**b**) ZX4, (**d**) ZX6 and (**f**) ZX8.

The as cast microstructures are shown in the Figure 1a,b,e, Figure 2a–c and Figure 3a,b, respectively for the alloys ZX4, ZX6, and ZX8. The phases present in these microstructures that were confirmed by XRD (Figure 4a,c,e) and quantitative analyses (Supplementary Tables S2–S4) are summarised in Table 1. Both the αNb_5_Si_3_ and βNb_5_Si_3_ were present in the as cast alloy ZX4 and only the βNb_5_Si_3_ in the as cast alloys ZX6 and ZX8 (Figure 4a,c,e and Table 1). There were Ti rich Nb_5_Si_3_ grains in all three alloys.

In the alloy ZX4, the most severe (strongest) macrosegregation was observed between the bottom and bulk of the button, i.e., between the parts that had experienced the highest and lowest cooling rates during solidification. The bottom was leaner in Si and the bulk leaner in Ti. In the same alloy, the C14-NbCr_2_ Laves phase was observed only in the bottom of the as cast button where it was formed in-between Nb_ss_ grains either as an individual phase or as part of a ternary eutectic (Figure 1b). Also, in the bottom, the Nb_ss_ + Nb_5_Si_3_ eutectic was less evident, and the Nb_ss_, Nb_5_Si_3_, and Nb_3_Sn phases were present as a co-continuous structure (Figure 1a). In the top and bulk, the Nb_5_Si_3_ and Nb_3_Sn were formed at a large volume fraction with Nb_ss_ + Nb_5_Si_3_ eutectic in inter-dendritic areas. 

In the alloy ZX6, the most severe macrosegregation was also observed between the bottom and bulk of the button, and the bottom was leaner in both Si and Ti. The microstructure consisted of the βNb_5_Si_3_, Nb_3_Sn, and Nb_ss_ phases (Figure 2a and Figure 4c and Table 1) with larger volume fractions of Nb_3_Sn and Nb_ss_ + βNb_5_Si_3_ eutectic in the bulk, and low volume fractions of the eutectic and the Nb_ss_ in the bottom of the button. A zone about 50 μm thick consisting of Nb_ss_ and βNb_5_Si_3_ with some Ti rich Nb_5_Si_3_ was formed next to the crucible wall (Figure 2b). Then, there was a change in microstructure to one consisting mainly of Nb_3_Sn and βNb_5_Si_3_ with a strong segregation of Ti in the latter (Figure 2c) and a very low volume fraction of Nb_ss_ and no Nb_ss_ + βNb_5_Si_3_ eutectic. The transition appeared to have started from a thin layer of uniform thicker Nb_ss_ that had formed parallel to the crucible wall (dotted line with short arrows in Figure 2b). The Nb_3_Sn was present at a higher volume fraction in the bottom of ZX6. The Ti concentration in the Nb_ss_ was high (Supplementary Table S3). 

In the alloy ZX8, there was macrosegregation of all elements with the exception of Sn. The strongest macrosegregation was observed between the bulk and top of the button and the highest concentration of Si was observed in the bulk. A very inhomogeneous microstructure in the bulk, top, and bottom of the alloy was formed owing to these differences in composition (Figure 3a–c). In contrast to the as cast microstructures of the alloys ZX4 and ZX6, no Nb_ss_ and no eutectic were observed in ZX8 (Figure 4e and Table 1) and the βNb_5_Si_3_, Nb_3_Sn, and C14-NbCr_2_ Laves phase were confirmed by XRD (Figure 4e) and microanalysis (Supplementary Table S4). The Nb_5_Si_3_ dendrites were larger in the bulk compared with the top and bottom of the button. The Laves phase was found throughout the alloy and formed very fine grains (Figure 3a).

### 4.2. Heat Treated Alloys

The actual compositions of the heat treated alloys are given in Table 1 and the microstructures are shown in the Figure 1c, Figure 2d and Figure 3d, respectively, for the alloys ZX4, ZX6, and ZX8. The phases that were confirmed by XRD (Figure 4b,d,f) and quantitative microanalyses (Supplementary Tables S2–S4) are summarised in Table 1. The αNb_5_Si_3_ and Nb_3_Sn were present in all three alloys and the solid solution was absent in the heat treated alloy ZX6. The eutectic microstructures were not stable in the alloys ZX4 and ZX6.

In the alloy ZX4 precipitates, about 3 μm in diameter, were observed within the αNb_5_Si_3_ (Figure 1d). Some of the Nb_ss_ grains were “surrounded” by a thin layer of Laves phase, as can be seen in the Cr X-ray map in Figure 1d. Inside some Nb_ss_ grains, Ti and nitrogen rich phase had also formed. The Sn and Si X-ray maps in Figure 1d suggest that the fine precipitates in αNb_5_Si_3_ were Nb_3_Sn. Contamination by nitrogen was also confirmed in the alloy ZX6 (Figure 4d) as well as Sn rich precipitates in αNb_5_Si_3_ grains (Figure 2d,e). In the alloy ZX8, the Nb_ss_ was formed together with αNb_5_Si_3_ and Nb_3_Sn (Figure 3d and Table 1) and in a few αNb_5_Si_3_ grains there were fine precipitates that exhibited contrast similar to that of the Nb_ss_ and Nb_3_Sn. The Nb_ss_ was rich in Cr (9.2 at.%) and Al (6.0 at.%), its Si concentration was 0.6 at.% and the Si/Sn ratio was 0.33. The Ti rich areas of the Nb_5_Si_3_ became richer in Si and leaner in Sn compared with the as cast alloy and the Si + Al + Sn concentration increased and was closer to the stoichiometric composition of unalloyed Nb_5_Si_3_. The Nb_3_Sn was leaner in Ti after heat treatment.

### 4.3. Oxidation

#### 4.3.1. Thermogravimetric (TG) Analysis and Oxidation Kinetics

**Figure 5:**
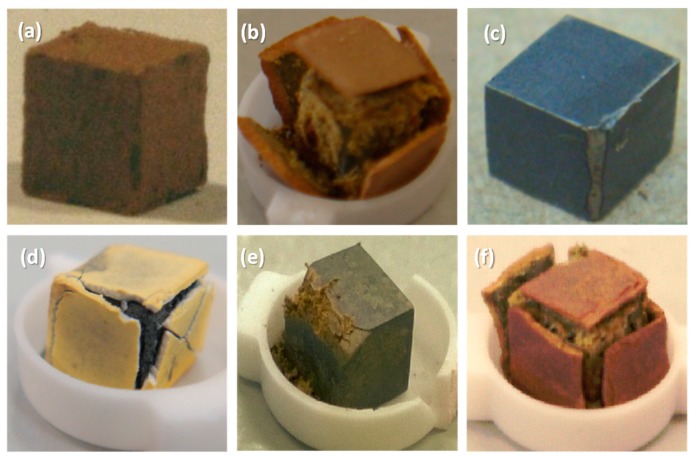
Images of specimens after isothermal oxidation at 800 °C (**a**,**c**,**e**) and 1200 °C (**b**,**d**,**f**). Alloys ZX4 (**a**,**b**), ZX6 (**c**,**d**) and ZX8 (**e**,**f**). Samples are approximately 3 × 3 × 3 mm^3^ before oxidation.

**Figure 6:**
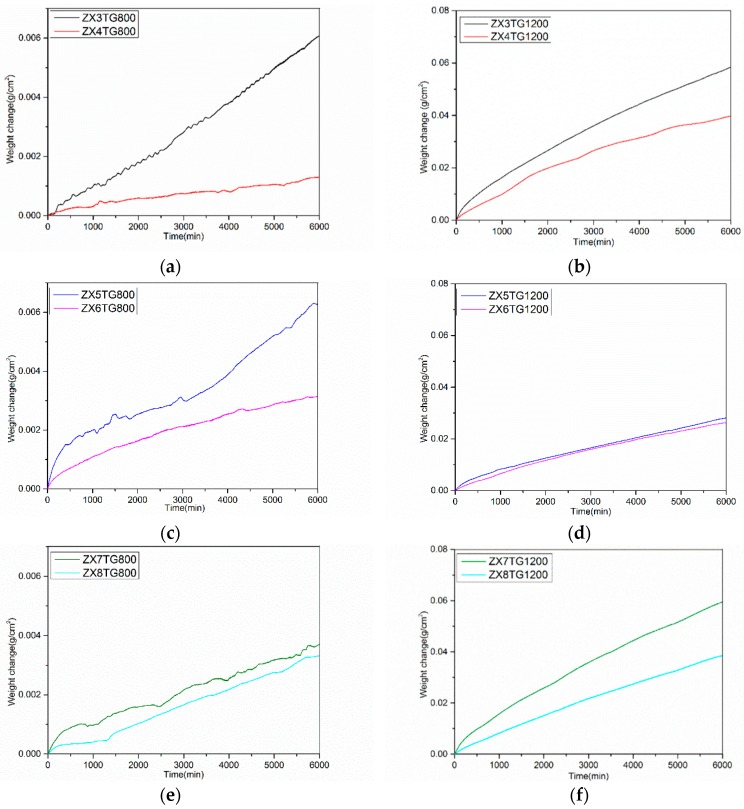
TG data for the isothermal oxidation at 800 °C (**a**,**c**,**e**) and 1200 °C (**b**,**d**,**f**) including the data for the alloys ZX3, ZX5 and ZX7 with 2 at.% Sn addition [13] for comparison purposes. Alloys ZX3, ZX4 (**a**,**b**), ZX5, ZX6 (**c**,**d**) and ZX7, ZX8 (**e**,**f**).

The specimens after isothermal oxidation at 800 and 1200 °C are shown, respectively, in (Figure 5a,c,e) and (Figure 5b,d,f). In Figure 6, where the weight gain with time is shown, the data for the low Sn content alloys (2 at.%) from [13] is included to show the effect of an increase in Sn concentration in the alloys. At 800 °C, all three alloys gained less weight than the 2 at.% Sn content alloys (Figure 6a,c,e), did not pest, and formed adherent oxides on their surfaces. At 1200 °C the weight gain of all three alloys was more severe than at 800 °C (Figure 6b,d,f), and there was spallation of the scales (Figure 5b,d,f). Those scales were between 250 and 500 μm thick. The weight gains of the alloys ZX4 and ZX8 were lower than the equivalent alloys with only 2 at.% of Sn (ZX3 and ZX7) (Figure 6b,f) and for the alloy ZX6, the weight gain difference from the equivalent alloy with 2 at.% Sn (ZX5) was very small (Figure 6d). Figure 5d suggests better adhesion of the scale that formed on ZX6 at 1200 °C compared with the alloys ZX4 and ZX8.

The oxidised specimen of the alloy ZX8 at 800 °C showed severe attack along one of its edges, but not on the other surfaces that were less severely oxidised compared with the alloy ZX7 with 2 at.% Sn addition. The Nb_ss_ is the Achilles’ heel in the oxidation of Nb-silicide based alloys, and alloys with low vol.% Nb_ss_ have low toughness. There was no Nb_ss_ in the specimen of ZX8. The specimen shown in Figure 5e does not represent the inherent oxidation resistance of the alloy ZX8 at 800 °C. The severe attack along one of its edges was attributed to pre-existing cracks in the specimen that were either present owing to the severe macrosegregation of this alloy and the absence of Nb_ss_, and cracks that were most likely formed during specimen preparation. 

The weight gains and oxidation rate constants of the alloys after isothermal oxidation at 800 and 1200 °C are given in Table 3. At 800 °C, the alloy ZX4 gained less weight than the other two alloys and followed parabolic kinetics during 100 h of oxidation while the oxidation of the alloys ZX6 and ZX8 was parabolic in the early stages and linear thereafter. At 1200 °C the weight gain of the alloy ZX6 was the lowest and those of the alloys ZX4 and ZX8 were essentially the same. The alloys ZX6 and ZX8 followed linear kinetics during 100 h oxidation, but the oxidation of the alloy ZX4 was parabolic in the early stages and linear thereafter. 

#### 4.3.2. Microstructures after Isothermal Oxidation at 800 °C

**Figure 7:**
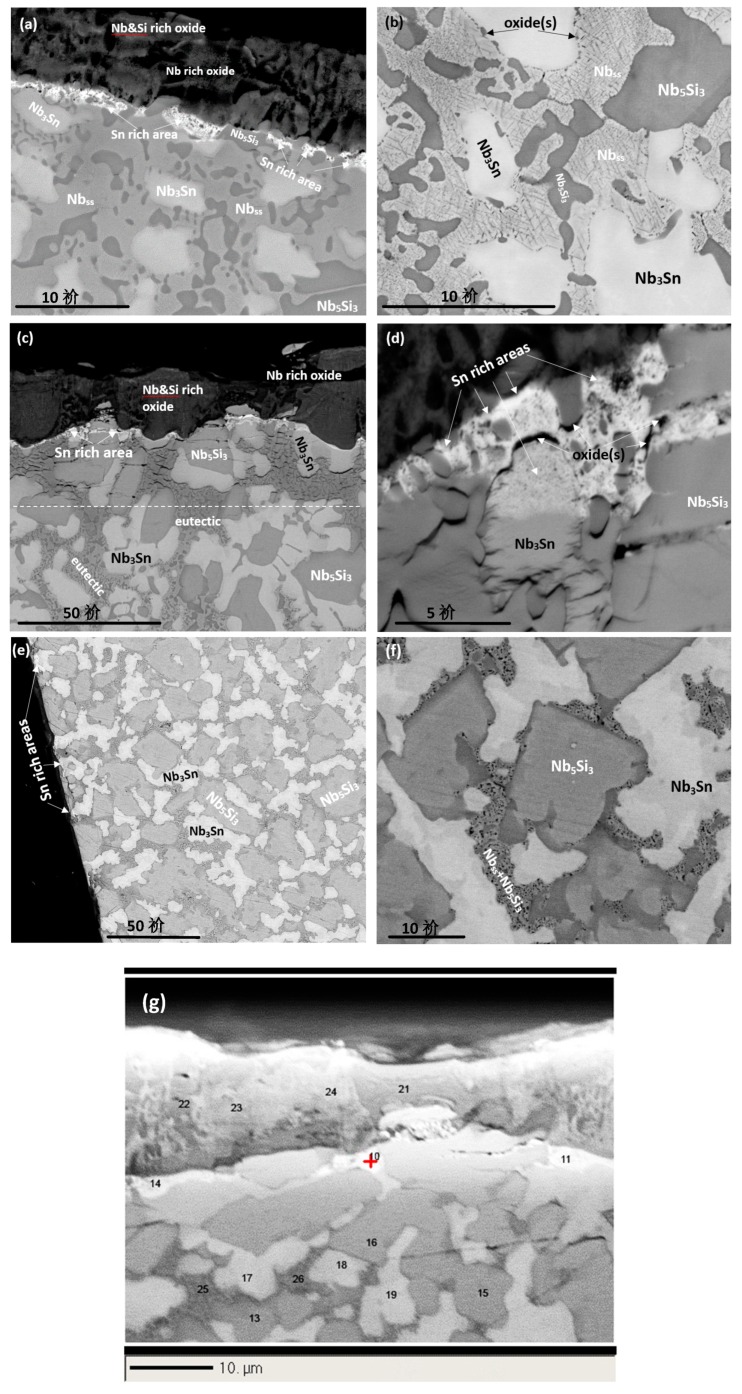
BSE images of cross sections of the oxidised specimens of the alloys ZX4 (**a**,**b**), ZX6 (**c**,**d**) and ZX8 (**e**,**f**) at 800 °C. The numbers in (**g**) indicate the “spots” of WDS analyses in the Sn rich area and near it (see Table 5). The dashed line in (**c**) indicates the depth of the diffusion zone.

The alloy ZX4 formed a compact and well adhering scale. A Sn rich area had formed between the scale and diffusion zone (Figure 7a). Alike the low Sn alloy ZX3, the Sn rich area exhibited a variety of contrasts, but different from ZX3 the Sn rich area not only formed continuously between the scale/Nb_ss_ interface, but also formed at the scale/Nb_3_Sn interface. In the diffusion zone, contamination by oxygen occurred via the Nb_ss_. Dark contrast precipitates were formed within the Nb_ss_ and at its grain boundaries, and no new phases were observed in the Nb_5_Si_3_ and Nb_3_Sn intermetallics (Figure 7b). All three phases were contaminated by oxygen in the bulk of the alloy, as illustrated in Table 4.

A cross section of the alloy ZX6 is shown in Figure 7c. The scale was less porous than that of the low Sn alloy ZX5 and its thickness was about 10 μm, similar with the alloy ZX4. There were cracks in the Nb_5_Si_3_ and Nb_3_Sn intermetallics that were mostly parallel to the scale/substrate interface and appeared to be more severe in the Nb_5_Si_3_ than in the Nb_3_Sn. The depth of the diffusion zone, which is shown by the dashed line in Figure 7c, was about 30 μm, smaller than that of the low Sn alloy ZX5. Considering the contrast of phases in the diffusion zone and bulk in Figure 7c, both the prior eutectic and the Nb_3_Sn exhibited darker contrast in the diffusion zone, but the contrast of the Nb_5_Si_3_ grains was the same in the two areas. The contamination of the microstructure by oxygen had progressed along the Nb_ss_. 

Similar to the alloy ZX4, a Sn rich area exhibiting a variety of contrasts was formed between the scale and the diffusion zone of ZX6 (Figure 7c,d). Strong enrichment in Sn was observed over and/or near the Nb_ss_. There was also Sn enrichment in parts of some of the Nb_3_Sn grains that were near the scale/substrate interface (Figure 7d) as well as oxide(s). The latter exhibited dark contrast. The numbers of WDS spot analyses in the scale and the Sn rich area below it are shown in the Figure 7g and the data are summarised in Table 5. The latter shows (i) that niobates rich in Ti or Si were formed in the scale, (ii) that the Sn rich area was contaminated by oxygen and had Si + Al + Sn content in the range 24 to 30 at.%, i.e., within the solubility range of Nb_3_Sn in the Nb-Sn binary [21], (iii) that the contamination by oxygen was reduced significantly at a depth of about 20 μm below the scale, (iv) that the Nb_5_Si_3_ was less severely contaminated by oxygen compared with the Nb_3_Sn, and (v) that the prior eutectic was more severely contaminated than the Nb_3_Sn and Nb_5_Si_3_, owing to the presence of the Nb_ss_. 

In the bulk of ZX6, the Nb_5_Si_3_ in the prior eutectic was contaminated by oxygen, probably owing to it neighbouring the Nb_ss_. The Ti concentration in the Nb_5_Si_3_ was lower than that in the as cast microstructure. The Nb_5_Si_3_ was poorer in Ti near the centre of the grains. A “ring” of darker contrast surrounded some Nb_5_Si_3_ grains. The WDS analysis data for the Nb_5_Si_3_, Nb_3_Sn and the prior eutectic is given in Table 6. The average chemical composition of the Nb_3_Sn and Nb_5_Si_3_ below the scale (Table 5) was essentially the same as that of the same phases in the contaminated eutectic areas in the bulk (Table 6). It should be noted that it was possible to distinguish the contamination by oxygen of the prior eutectic areas in the bulk only in BSE mode and at high magnification. 

A cross section of the alloy ZX8 is shown in Figure 7e. A thin scale was formed on its surface with a thin Sn rich area between the scale and Nb_3_Sn, which is indicated by arrows in Figure 7e. The diffusion zone was also thin (about 10 μm) and in this zone the Nb_3_Sn and Nb_5_Si_3_ were cracked. The bulk was contaminated by oxygen and a eutectic like Nb_ss_ + Nb_5_Si_3_ microstructure was observed (Figure 7f), which was not present in the as cast alloy. The Nb_5_Si_3_ in the bulk had Ti rich areas that exhibited darker contrast. This was also the case for the Nb_3_Sn phase that was enriched in Ti at the edge of grains and showed a slightly darker contrast. The oxygen concentration in the Nb_5_Si_3_ and Nb_3_Sn phases in the diffusion zone was low (Table 7) compared with that in the Nb_ss_ in the low Sn alloy ZX7 [13]. The WDS analysis of the phases in the bulk microstructure is shown in Table 8. The Laves phase, which was observed in the as cast and heat treated alloy ZX8, was present in the bulk of the oxidised alloy, but its analysis was not possible owing to its small size and a contrast similar to that of the Ti rich Nb_5_Si_3_. Its presence was confirmed by the very Cr rich areas in X-ray maps (not shown).

#### 4.3.3. Microstructures after Isothermal Oxidation at 1200 °C

**Figure 8:**
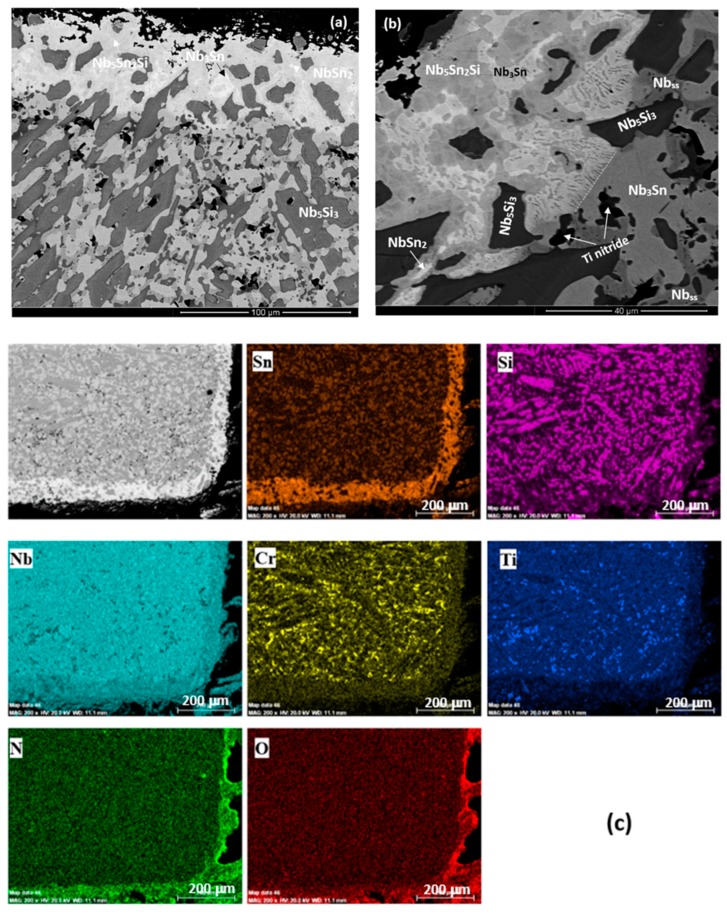
(**a**,**b**) BSE images of a cross section of the oxidised alloy ZX4 at 1200 °C and (**c**) X-ray maps showing the continuous Sn rich area that formed around the specimen. (**b**) shows details of the micro-structure in the Sn rich area. For dashed line in (**b**) see text.

The cross section of the alloy ZX4 in Figure 8a,c shows a continuous Sn rich area significantly thicker than that at 800 °C. Details of this zone can be seen in Figure 8b and the distribution of elements is shown in the X-ray maps in Figure 8c. The vol.% of Nb_5_Si_3_ grains in the Sn rich area was lower compared with the bulk. Ti nitride was formed at a lower volume fraction compared with the low Sn alloy ZX3 (Figure 8b). The chemical compositions of phases in the Sn rich area are shown in Table 9. In the bulk, Ti nitrides and oxides were formed at the grain boundaries of Nb_ss_. The oxides were rich in Cr and O according to X-ray maps (not shown). Both the Nb_5_Si_3_ and Nb_3_Sn were contaminated by oxygen in the bulk (Table 10).

**Figure 9:**
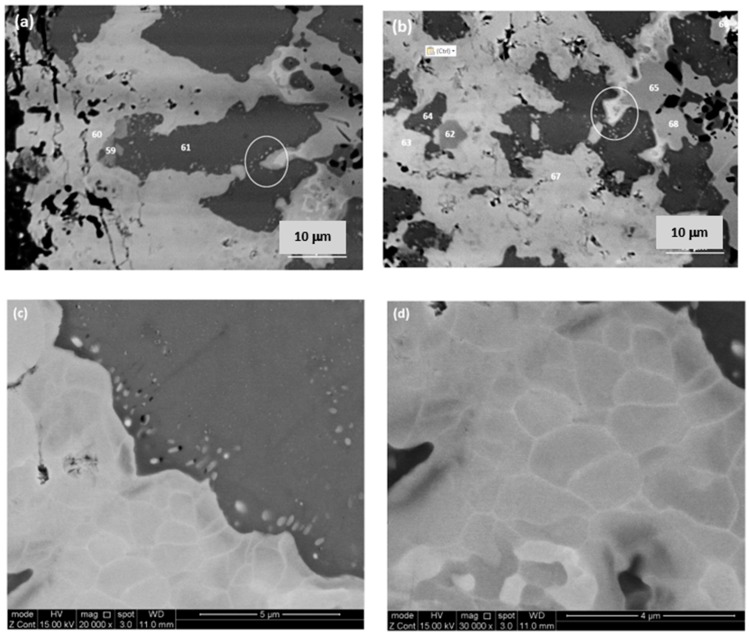
(**a**,**b**) BSE images of the microstructure of the Sn rich area of the alloy ZX6 at 1200 °C with numbers indicating the WDS spot analyses. For the analysis data, see Table 11. (**c**,**d**) show details of the microstructure indicated by circles in (**a,b**).

A continuous, about 50-μm thick, Sn rich area like the one observed in the alloy ZX4 was also formed in the alloy ZX6. In this case however, this area had noticeably more cracks parallel to the scale/substrate interface that were not confined only in the Nb_5_Si_3_. In parts of the cracked Sn rich area, Nb_5_Si_3_ and Nb_3_Sn grains were present, but not the Nb_ss_. In contrast to the alloy ZX4, Ti nitrides were present throughout the cross-section microstructure but not in the Sn rich area. The X-ray maps showed (i) Ti nitride below the Sn rich area but not in it, (ii) that Sn rich phase(s) were formed around Nb_5_Si_3_ grains, and (iii) that the Al concentration in the Sn rich area was lower than that below it and in the Nb_5_Si_3_ grains. The WDS analysis data of the Sn rich area are summarised in Table 11 and the numbers of the spot analyses are shown in Figure 9. In the Sn rich area three phases were observed. All phases had been contaminated by oxygen. Analyses 60, 63, and 67 were from the major phase in the Sn rich area for which the Si + Al + Sn concentration was around 35 at.%, the Sn concentration between 21 and 24 at. %, Si from 8 to 14 at.% and Al in the range 0.6 to 1.8 at.%. The Nb_5_Si_3_ phase in the Sn rich area (analyses 59, 61, 62, 64) had Si + Al + Sn content around 37 at.%, with Si in the range 30.5 to 33 at.%, Sn in the range 1.8 to 6.7 at.% and Al in the range 0.3 to 2 at.%. The Nb_3_Sn (analyses 65,66) was more heavily contaminated by oxygen than the Nb_5_Si_3_. The composition of the Nb_3_Sn adjacent to the Sn rich area (analyses 65,68) agreed with the composition in the bulk, see Table 12. The Nb_5_Si_3_ in the bulk was leaner in Sn compared with that of the silicide in the Sn rich area.

**Figure 10:**
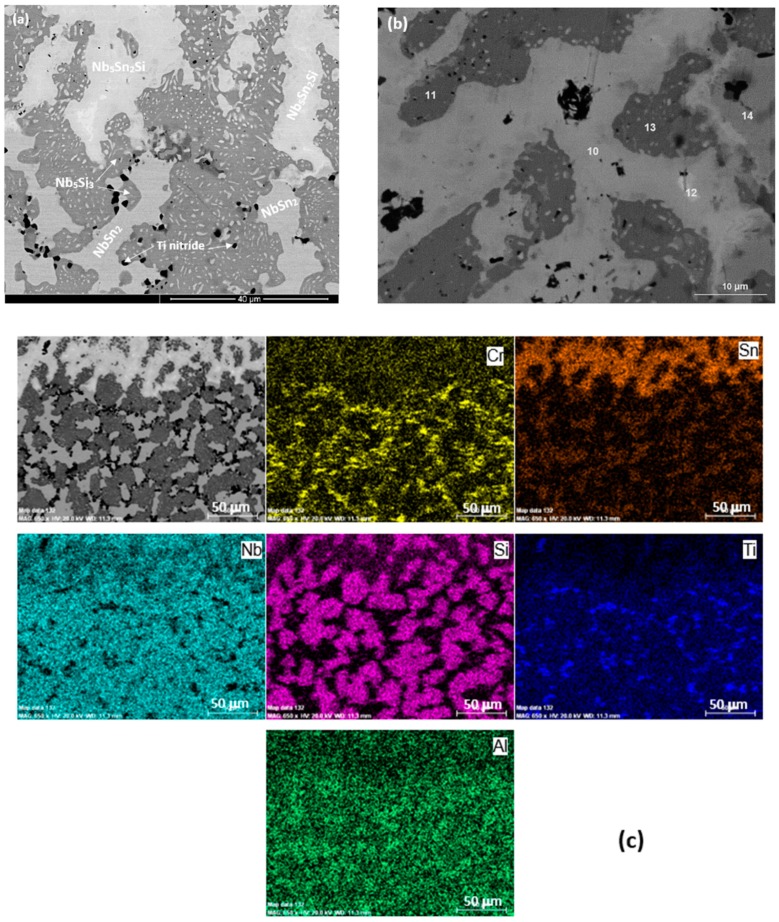
(**a**,**b**) BSE images and (**c**) X-ray elemental maps of the Sn rich area in the alloy ZX8 at 1200 °C.

The alloy ZX8 also formed a continuous, about 50-μm thick, Sn rich area like the alloys ZX4 and ZX6. This area was not severely cracked. Details of the Sn rich area are shown in Figure 10a. The Laves phase was not observed in the Sn rich area but was present in the bulk (Figure 10c). Ti nitrides were formed in the Sn rich area (Figure 10a) and in the bulk. The WDS analysis data for phases in the Sn rich area is given in Table 13 and the spot analysis numbers are shown in the Figure 10b. Analysis 10 corresponds to Nb_5_Sn_2_Si. Analyses 11 and 13 correspond to Nb_5_Si_3_. The higher Sn concentration in analysis 11 was attributed to the enrichment of the silicide with Sn. Analysis 12 shows a very rich in Sn phase with 40 at.% Sn. The size of this phase however was very small and it is likely that the analysis was influenced by the surrounding phase (analysis 10), which was also rich in Sn. Towards the bulk, the Nb_3_Sn was present (analysis 14). The WDS analysis data for the Nb_5_Si_3_ and Nb_3_Sn in the bulk is shown in Table 14. Both phases were contaminated by oxygen. The Laves phase was confirmed by X-ray maps (not shown) and was rich in Si.

#### 4.3.4. Scales at 800 and 1200 °C

**Figure 11:**
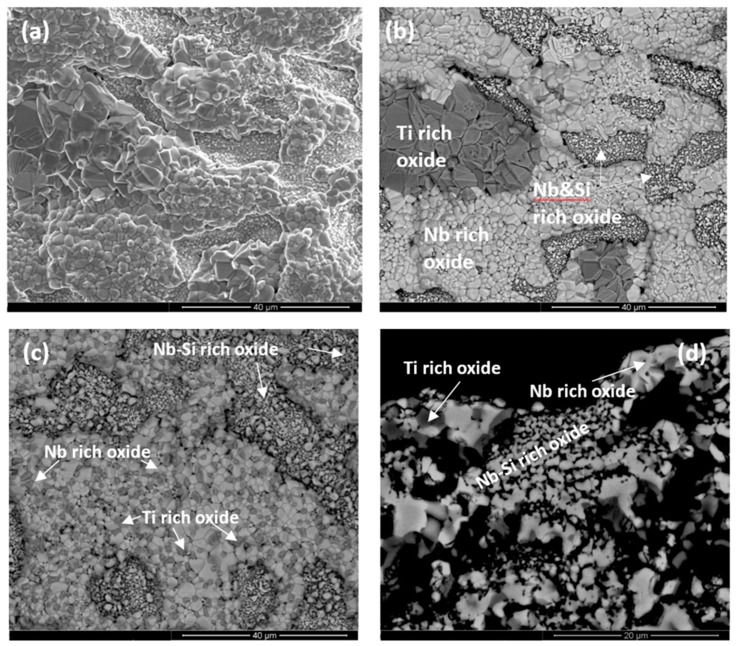

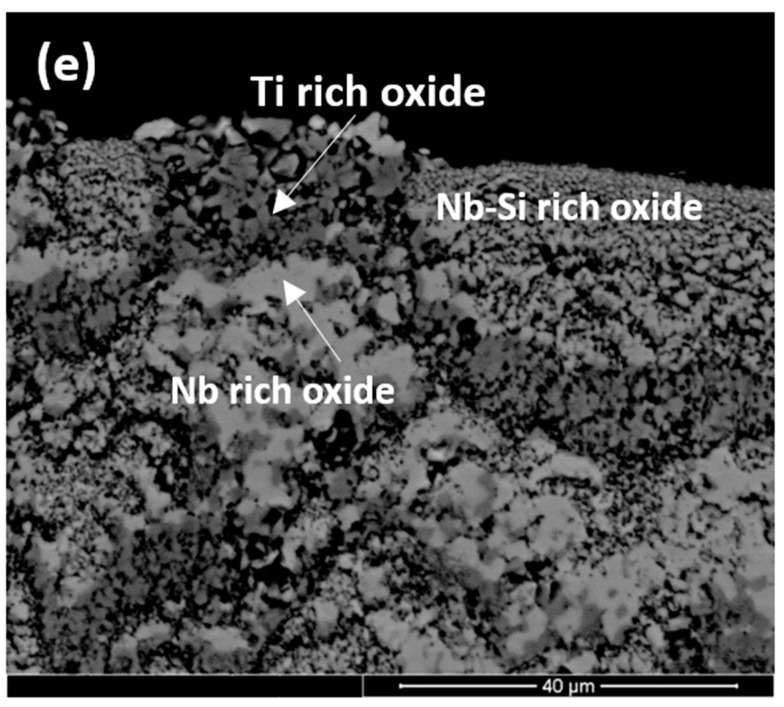
Images of the scales formed on the alloys ZX4 (**a**,**b**), ZX6 (**c**,**d**) and ZX8 (d) at 1200 °C. (**a**) Secondary electron image, (**b**–**e**) BSE images. (**a**–**c**) show topology of scales, and (**d**,**e**) are cross sections of scales.

Images of the scales formed on the alloys at 1200 °C are shown in the Figure 11 and WDS analysis data of oxides in the scales that formed at 800 and 1200 °C is given in Table 15. Data for the oxides in the scale of the alloy ZX6 at 800 °C was given in the Table 5. At 800 °C the scale consisted of Nb rich and Nb and Si rich oxides in all three alloys. Ti rich oxide was also observed in the scale of the alloy ZX4 at 800 °C but WDS analysis of its chemical composition was not possible. At 1200 °C, Nb rich, Nb and Si rich, and Ti-rich oxides were formed in all three alloys.

## 5. Discussion

### 5.1. Macrosegregation

There was macrosegregation in all three as cast alloys (Table 2). Phenomena linked with the macrosegregation of elements in Nb-silicide based alloys were discussed in [20] where the macrosegregation of Si was linked with the partitioning of other solutes between the key phases in the microstructure of Nb-silicide based alloys, namely the Nb_ss_, Nb_5_Si_3_, C14-NbCr_2_ Laves and the Nb_ss_ + Nb_5_Si_3_ eutectic [16,17,18,19]. Tin is one of the alloying additions that have a strong effect on macrosegregation [20]. The alloy design methodology NICE [14] predicts higher Si macrosegregation in Nb-silicide based alloys of the Nb-Ti-Si-Al-Cr-Sn system as the Sn concentration is increased (see Section 2). In [20], the ranking of Nb-silicide based alloys in terms of increasing Si macrosegregation indicated that the latter tended to increase when the parameters ΔH_m_/T_m_ (“alloys entropy of fusion”), T_m_^sp^ (melting temperature of sp electronic configuration elements), and [ΔH_m_/T_m_][ΔH_m_^sd^/ΔH_m_^sp^]^−1^ increased and the ratios ΔH_m_^sd^/ΔH_m_^sp^ and T_m_^sd^/T_m_^sp^ and the parameters ΔH_m_ (“alloy enthalpy of melting”), T_m_ (alloy melting temperature), and T_m_^sd^ (melting temperature of the sd electronic configuration elements) decreased. According to [20], the potency of the parameters T_m_, ΔH_m_, T_m_^sd^, ΔH_m_^sd^/ΔH_m_^sp^, and T_m_^sp^ is strong and that for the other parameters is weak.

The alloy ZX4 can be compared with the alloy Nb-24Ti-18Si-5Sn (alloy NV6 in [22]) and Nb-24Ti-18Si-5Cr (alloy KZ4 in [23]). In the as cast alloy NV6, the microstructure consisted of the Nb_5_Si_3_ (primary phase), Nb_ss_, Nb_3_Sn, and Nb_ss_ + Nb_5_Si_3_ eutectic with strong partitioning of Ti in the Nb_5_Si_3_ and strong macrosegregation of Si and Ti (MACSi and MACTi 5.5 and 8.4 at.%, respectively). In the as cast alloy ZX4, the microstructure consisted of Nb_5_Si_3_ (primary phase), Nb_ss_, Nb_3_Sn, a very low volume fraction of Nb_ss_ + Nb_5_Si_3_ eutectic, and a very low volume fraction of C14-NbCr_2_ Laves phase, some of which participated in a ternary eutectic in the bottom of the button. There was strong macrosegregation of Si and Ti (MACSi and MACTi 7.3 and 6.6 at.%, respectively). In both alloys (i.e., NV6 and ZX4) the Nb_5_Si_3_ was present in its β and α types. In the Nb_ss_ the Si+Sn content was 7.7 and 9.2 at.% and the ratio Si/Sn was 0.3 and 0.64, respectively in the two alloys. The addition of Cr did not eliminate the macrosegregations of Si and Ti but reversed their severity. The addition reduced the partitioning of Ti in Nb_5_Si_3_ (allowing more Ti in the last to solidify melt), reduced significantly the fraction of the Nb_ss_ + Nb_5_Si_3_ eutectic, and made possible the formation of the C14-NbCr_2_ Laves phase in the last to solidify melt and the ternary eutectic in the bottom of the button of ZX4, which was also richer in Cr compared with the top and bulk. Considering the macrosegregation of Si, the alloy ZX4 is compared with the Nb_3_Sn containing alloys NV6 and Nb-18Si-5Sn (alloy NV9 in [22]) in Table 16. For all four alloys, there is good agreement with the trends discussed in [20] for the parameters ΔH_m_/T_m_, ΔH_m_^sd^/ΔH_m_^sp^, T_m_^sd^/T_m_^sd^, [ΔH_m_/T_m_] × [ΔH_m_^sd^/ΔH_m_^sp^]^−1^, shown by the arrows and bold numbers in the table. For the Nb_3_Sn containing alloys NV9, NV6, and ZX4, there is also good agreement with [20] for the parameters ΔH_m_, T_m_, and T_m_^sd^. 

In the case of the alloys ZX5 [13] and ZX6, the macrosegregation of Si was essentially the same considering the error for Si analysis. This makes it difficult to “rank” the two alloys using the aforementioned parameters. If we were to assume that the trends reported in [20] and confirmed for the macrosegretation of Si in the alloy ZX4 apply also in the case of the alloys ZX5 and ZX6, we can compare these two alloys with the alloy Nb-24Ti-18Si-5Al (alloy KZ7 in [23]) (see Table 17). The ranking of the alloys in the latter shows that the trends are followed for all the paremeters (shown by the arrows and bold numbers) with the exception of the parameter ΔH_m_. 

In Table 17, the macrosegregation of Si in the alloy ZX6 is also compared with the alloys NV9 and NV6 (Nb-24Ti-18Si-5Sn) [22]. All three alloys had the same phases in their micro-structures, namely Nb_ss_, Nb_5_Si_3_, and Nb_3_Sn. Given that the Si macrosegregation in the alloys NV6 and ZX6 was essentially the same, the same assumption as above was made. The data in Table 17 show that the trends reported in [20] are followed by the parameters (shown by the arrows and bold numbers), with the exception of the parameter T_m_^sp^. It should be noted that the same was the case for the alloys ZX4, NV6, and NV9 (see Table 16).

In the alloy ZX8, the macrosegregation of Si was marginally more severe compared with that of Ti (MACSi and MACTi 10 and 9.7 at.%, respectively, see Table 2). The macrosegregation values of both elements were the highest measured among the three alloys studied in this paper. The severe macrosegregation in ZX8 resulted in highly inhomogeneous microstructures. Table 18 shows that the parameters ΔH_m_, T_m_, ΔH_m_^sd^/ΔH_m_^sp^, T_m_^sd^ and T_m_^sd^/T_m_^sp^ can describe the macrosegregation of Si. If the data for the alloy ZX6 were to be included in the comparison of alloys in Table 18, then the ranking shown in the latter is destroyed. This would suggest that the parameters used in Table 18 cannot capture how the synergy of Al with Sn and Cr in an alloy affects the macrosegregation of Si. Another reason for this could be that the potency of material parameter(s) changes caused by a particular element when in synergy with other additions in Nb-silicide based alloys is different for Sn, and Al and Cr. The potency is strong regarding ΔH_m_, ΔH_m_/T_m_, and T_m_^sp^ for Sn and T_m_, ΔH_m_/T_m_, ΔH_m_^sd^/ΔH_m_^sp^, T_m_^sd^, T_m_^sp^, and T_m_^sd^/T_m_^sp^ for both Al and Cr, but weak for Sn regarding the parameters T_m_, T_m_^sd^ and T_m_^sd^/T_m_^sp^ (see Table 3 in [20]). It is interesting that trends are followed by the latter three parameters in Table 18. 

### 5.2. Microstructures and Phase Stability

#### 5.2.1. Nb-24Ti-18Si-5Cr-5Sn (Alloy ZX4)

The microstructure in the top and bulk of the as cast alloy ZX4 consisted of Nb_5_Si_3_, Nb_ss_, Nb_3_Sn, and Nb_ss_ + Nb_5_Si_3_ eutectic. As the primary βNb_5_Si_3_ formed, the surrounding melt became rich in Cr, Sn and Ti and poor in Si (Supplementary Table S2). Because Sn has a strong effect on the partitioning of Ti in Nb_5_Si_3_ [17,22], the concentration of Ti in the melt near the Nb_5_Si_3_ dendrites differed. Nb_3_Sn formed next to the Ti rich Nb_5_Si_3_, making the surrounding melt richer in Cr and Si and poorer in Sn and next to the less Ti rich Nb_5_Si_3_ formed by the Nb_ss_ making the surrounding melt richer in Si and poorer in Cr and Sn. The Nb_ss_ + βNb_5_Si_3_ eutectic formed in the last to solidify melt. It is suggested that the solidification path in the bulk and top of ZX4 was L → L + βNb_5_Si_3_ → L + βNb_5_Si_3_ + Nb_3_Sn and/or L + βNb_5_Si_3_ + Nb_ss_ → L + βNb_5_Si_3_ + Nb_3_Sn + Nb_ss_ + (Nb_ss_ + βNb_5_Si_3_) eutectic. 

In the bottom of ZX4, the microstructure consisted of primary βNb_5_Si_3_, Nb_3_Sn, Nb_ss_, and C14-NbCr_2_ Laves phase and in some areas the last to solidify melt gave a ternary eutectic. The Nb_ss_ + βNb_5_Si_3_ eutectic formed some distance away from the water cooled crucible and as the Laves phase was not observed in the areas with the Nb_ss_ + βNb_5_Si_3_ eutectic, the transition, which is indicated by the dashed line in Figure 1e, must have occurred when the inter-dendritic melt became lean in Cr. The partitioning of Cr and other elements between Nb_ss_, Nb_3_Sn, and Nb_5_Si_3_ must have played a role in the transition from the bottom to the bulk microstructure.

The microstructures in Figure 1a,b show Nb_3_Sn surrounded by Nb_ss_. This was not observed in the bulk and top of the button of ZX4. Such a microstructure could be the product of the peritectic reaction L + Nb_3_Sn → Nb_ss_. The latter, however, does not exist in the Nb–Sn binary [21], where in the Nb rich side of the binary the peritectic reaction is L + Nb_ss_ → Nb_3_Sn. Thus, if the former reaction did indeed occur in ZX4, then it must have been promoted by the synergy of Sn with Cr and Ti in the solidification of the Cr richer melt under higher cooling rates (compared with the bulk) in the bottom of ZX4. There, as the primary βNb_5_Si_3_ formed, the surrounding melt became rich in Cr, Sn, and Ti and poor in Si. Because of the higher cooling rates, in the solidifying melt, the partitioning of Ti in Nb_5_Si_3_ was not as strong as in the bulk and top and thus the concentration of Ti in the melt near the Nb_5_Si_3_ dendrites was high. In Ti and Sn, rich inter-dendritic constitutionally undercooled melt formed the Nb_3_Sn (Ti_3_Sn is in equilibrium with the melt at a lower temperature than Nb_3_Sn, thus Ti (substituting Nb) in Nb_3_Sn is expected to depress this temperature, meaning less undercooling is required for the formation of the A15 intermetallic, and the surrounding melt became rich in Cr and Si and from this melt the Nb_ss_ formed via the aforementioned peritectic reaction. In Cr rich melt, the Laves phase subsequently formed and in inter-dendritic areas where the melt reached the ternary eutectic composition, the ternary eutectic formed. It is suggested that the solidification path in the bottom of ZX4 was L → L + βNb_5_Si_3_ then L + βNb_5_Si_3_ + Nb_3_Sn and L + βNb_5_Si_3_ + Nb_ss_ + Nb_3_Sn (in parts with L + Nb_3_Sn → Nb_ss_), then L + βNb_5_Si_3_ + Nb_3_Sn + Nb_ss_ + NbCr_2_ and finally L → βNb_5_Si_3_ + Nb_3_Sn + Nb_ss_ + NbCr_2_ + (βNb_5_Si_3_ + Nb_ss_ + NbCr_2_)_eutectic_.

The microstructure of the heat treated (1200 °C/100 h) alloy NV6 consisted of Nb_ss_, Nb_3_Sn, αNb_5_Si_3_ with Ti rich areas and coarsened prior eutectic [22]. The same phases plus Ti nitrides but with no Ti rich αNb_5_Si_3_ and no C14-NbCr_2_ Laves phase were observed in the heat treated (1500 °C/100 h) alloy ZX4. There were brighter contrast second phase precipitates in the bulk of αNb_5_Si_3_ grains in ZX4 that were not observed in NV6-HT. The absence of the C14-NbCr_2_ Laves phase and the presence of Ti nitrides were also confirmed in the heat treated (1500 °C/100 h) alloy KZ4 (Nb-24Ti-18Si-5Cr) [23]. In all three alloys (i.e., NV6, KZ4 and ZX4), the Si solubility in the Nb_ss_ was in the same range. In the Nb_3_Sn the Si+Sn content was 16.3 and 18.9 at.% and the ratio Si/Sn was 0.5 and 0.4, respectively, in the alloys NV6 and ZX4. In the Nb_ss_ the Si + Sn content was 4.3 and 5.6 at.% and the ratio Si/Sn was 0.1 and 0.14, respectively in the alloys NV6 and ZX4. In other words, the synergy of Cr with Sn had minor effects on the chemical composition of the Nb_3_Sn and Nb_ss_ and was not able to eliminate the contamination of the alloy ZX4 by nitrogen. 

#### 5.2.2. Nb-24Ti-18Si-5Al-5Sn (Alloy ZX6)

The βNb_5_Si_3_ formed in the as cast alloy ZX6 (Figure 4c), as well as in the as cast alloy KZ7 (Nb-24Ti-18Si-5Al) [23]. This provides further support to the conclusion of Zelenitsas and Tsakiropoulos [23] that Al stabilises the βNb_5_Si_3_ during solidification and the conclusion of the authors in [13] that the synergy of Al with Sn increases the sluggishness of the βNb_5_Si_3_ → αNb_5_Si_3_ transformation during solidification. It is suggested that the solidification path in the bulk and top of the cast alloy ZX6 was L → L + βNb_5_Si_3_ → L + βNb_5_Si_3_ + Nb_3_Sn → βNb_5_Si_3_ + Nb_3_Sn + (Nb_ss_ + βNb_5_Si_3_)_eutectic_. 

The average Si concentration in the bottom of the button (Supplementary Table S3) was lower than the “accepted” eutectic composition in the Nb–Si binary [24]. The microstructure that formed from the highly undercooled melt that solidified next to the water-cooled crucible was different (see Figure 2b). An area about 50-μm thick (we shall call this area A) was formed first and consisted of Nb_ss_ and Nb_5_Si_3_ with some Ti rich Nb_5_Si_3_ (circle in Figure 2b) forming away from the side that was in contact with the water cooled crucible (shown with dotted line and short arrows in Figure 2b) and before the transition, which is indicated by the dashed line in Figure 2b, to an area consisting mainly of Nb_3_Sn and Nb_5_Si_3_ (we shall call this area B). It is suggested that the formation of these two areas was associated with the strong macrosegregation of Si and Ti in the alloy ZX6 (Table 2). It is worth exploring how areas A and B were formed in the alloy ZX6, because similar transitions have been reported in an alumina scale forming complex concentrated alloy (or high entropy alloy) of the Nb-Ti-Si-Al-Hf system that was prepared using arc melting [25].

From the area B evolved the bulk microstructure. There was no normal eutectic (i.e., like the eutectic observed in the bulk and top of the as cast ZX6, Figure 2a) in area A and also in area B close to the Nb_ss_ layer forming the interface separating the two areas (Figure 2b). The entropies of fusion of the Nb, Nb_3_Sn, and Nb_5_Si_3_, respectively, are 9.45, 11.6 and 14.55 J/molK, meaning the Nb_5_Si_3_ silicide has the “greater difficulty for growth” (or needs more kinetic undercooling) than the Nb_3_Sn and the solid solution. In the alloy ZX6, three phases can form, namely Nb_ss_, Nb_5_Si_3_, and Nb_3_Sn. In area A, the latter was not observed. Considering that the primary phase is that which grows at the highest interface temperature (highest interface temperature criterion), the formation of Nb_ss_ instead of Nb_3_Sn would require the melt that solidified in area A to be poorer in Si + Al + Sn compared with that in area B. We suggest that this was the case owing to the strong macrosegregation in the alloy ZX6.

After the nucleation of the Nb_ss_ and βNb_5_Si_3_ on the crucible wall from the highly undercooled melt (notice the larger patches of Nb_ss_ (small arrows in Figure 2b) and the comparatively smaller patches of Nb_5_Si_3_ formed next to the crucible wall) the Nb_ss_ grew faster than (i.e., outgrew) the βNb_5_Si_3_ in the area A. As the Nb_ss_ grew, the surrounding melt became rich in Si and poor in Ti, Al, and Sn. As the βNb_5_Si_3_ grew, the surrounding melt became poor in Si and rich in Al, Sn, and Ti. In some parts of the area A, the melt became very rich in Ti and Si and some Ti rich Nb_5_Si_3_ formed in these parts of area A from the very Ti rich melt (circles in Figure 2b,c). 

As the macroscopic S/L interface advanced towards the interface with area B (and the melt undercooling decreased) and solutes partitioned between the Nb_ss_, Nb_5_Si_3_, and the melt, the latter became rich in Ti and poor in Si ahead of Nb_5_Si_3_ (rectangle in Figure 2c) and a continuous Nb_ss_ layer formed from this melt (Figure 2c). After some growth of the Nb_ss_ in the aforementioned continuous layer, Nb_5_Si_3_ started to form (small patches of Nb_5_Si_3_ are indicated by short arrows in Figure 2c). Ahead of this macroscopic interface (forming the boundary between areas A and B) that consisted mainly of Nb_ss_ with some Nb_5_Si_3_, the melt became richer in Si + Al + Sn (it is suggested that this was possible because (i) of solidification conditions in area A and (ii) the strong macrosegregation in ZX6) and in this melt, the growth of Nb_3_Sn instead of Nb_ss_ became possible (highest interface temperature criterion). The Nb_3_Sn grew faster than the Nb_5_Si_3_ owing to its lower entropy of fusion (see above). As the Nb_3_Sn grew, the surrounding melt became richer in Si and poorer in Sn, and as the Nb_5_Si_3_ grew, the surrounding melt became richer in Ti, Al, and Sn. There was also partitioning of Ti in the Nb_5_Si_3_ (Figure 2a), (meaning the melt near the Ti rich Nb_5_Si_3_ became poorer in Ti compared with the melt near the “normal” Nb_5_Si_3_) and after some distance from the macroscopic interface, formation of some Nb_ss_ was possible (Figure 2b,c). The above could explain why the volume fraction of Nb_ss_ in the bottom of ZX6 was lower compared with the bulk and top.

After the heat treatment, only two phases were observed, namely αNb_5_Si_3_ and Nb_3_Sn (Figure 4d), and there were still Ti rich areas in the Nb_5_Si_3_ (Supplementary Table S3). In other words, in the heat-treated microstructure, the Nb_ss_ was not stable, which would suggest that the synergy of 5 at.% Sn with Al and Ti in the alloy ZX6 had destabilised the Nb_ss_. The two phase microstructure is in agreement with the 1400 °C and 1500 °C isothermal sections of the Nb-Si-Al system proposed by Brukl et al. [26] and Pan et al. [27], if in the composition of the heat treated alloy (Table 1) the Al and Sn are considered to be equivalent, but not if the Si and Sn are considered as equivalent. Given the important role that Nb_ss_ plays in oxidation, toughness, and high temperature strength, the results for the alloy ZX6 show that the synergy of Al and Sn could be used to control the vol.% of Nb_ss_ in Nb-silicide based alloys (see next section).

#### 5.2.3. Nb-24Ti-18Si-5Al-5Cr-5Sn (Alloy ZX8)

The microstructure of the as cast alloy ZX8 would suggest that the solidification path in all areas of the button was L → L + βNb_5_Si_3_ → L + βNb_5_Si_3_ + Nb_3_Sn → βNb_5_Si_3_ + Nb_3_Sn + C14-NbCr_2_ with the strong segregation of Cr, Si, and Ti. The βNb_5_Si_3_ and Nb_3_Sn formed a co-continuous microstructure with the Laves phase forming in some parts in-between these two phases. As solidification progressed from the bottom to the top of the button, the melt became richer in Cr (Supplementary Table S4).

In the alloy ZX8, the formation of the Laves phase starved the melt from the Cr needed for the formation of the Nb_ss_. As a result of the suppression of the Nb_ss_ in all parts of the button, the Nb_ss_ + βNb_5_Si_3_ eutectic was not formed even though this eutectic was present in the lower Sn content alloy ZX7 [13], where the Laves phase was not observed. In the alloys ZX4 and ZX6 the vol.% of the Nb_ss_ + βNb_5_Si_3_ eutectic was reduced significantly compared with the lower Sn content alloys ZX3 and ZX5 [13]. The Nb_ss_ was also suppressed in the as cast alloy EZ7 (Nb-18Si-5Al-5Sn [28]) where a Nb_3_Sn + Nb_5_Si_3_ eutectic was formed. In this alloy the Nb_ss_ was not stable (not observed after heat treatment (1500 °C/100 h)). The Nb_ss_ was stable in the alloy NV9 (Nb-18Si-5Sn [22]), where also the Nb_ss_ + Nb_5_Si_3_ eutectic was formed. These facts would suggest (a) that the Nb_ss_ is not stable in alloys where Al and Ti are in synergy with 5 at.% Sn (data for the alloys ZX6 and EZ7 [28]), (b) that the synergies of Al or Cr individually with Ti and Sn destabilise the Nb_3_Sn + Nb_5_Si_3_ eutectic that is replaced by the Nb_ss_ + Nb_5_Si_3_ eutectic (alloys ZX3 [13], ZX4, ZX5 [13], ZX7 [13], versus EZ7 [28]), the formation of which is not completely suppressed even with a higher Sn concentration in the alloy (ZX4 and ZX6 versus ZX3 and ZX5 [13]), and (c) that the synergy of Al and Cr simultaneously with Ti and 5 at.% Sn suppresses the formation of the Nb_ss_ + Nb_5_Si_3_ eutectic in the cast microstructure (ZX8) but not the stability of the Nb_ss_ (ZX8). 

As the primary βNb_5_Si_3_ formed, the surrounding melt became richer in Al, Cr, Sn, and Ti and leaner in Si, while the melt next to Ti rich Nb_5_Si_3_ became richer in Al, Cr, and Sn and leaner in Si and Ti (Supplementary Table S4). In this melt, Nb_3_Sn was formed instead of Nb_ss_ according to the highest interface temperature criterion. With Nb_5_Si_3_ and Nb_3_Sn in the solidifying melt, the growth of the latter phase was kinetically easier owing to its lower entropy of fusion (see above). As the Nb_3_Sn was formed, the surrounding melt became richer in Cr and Si and leaner in Sn and Ti. From the growth of the Nb_5_Si_3_, Ti rich Nb_5_Si_3_, and Nb_3_Sn the melt became rich in Al and Cr, both of which stabilise the Laves phase, and in the Si containing and Al and Cr rich melt the C14-NbCr_2_ Laves phase formed. In the case of the cast alloy ZX4, where the Laves phase was observed only in the bottom of the button with Nb_ss_, Nb_5_Si_3_ and Nb_3_Sn, the Nb_ss_ was very rich in Cr (about 11.3 at.%) and the Laves was absent when the eutectic formed. In this alloy (ZX4) the Cr in the melt was consumed by the formation of the Nb_ss_ (and the Laves could not form). 

After heat treatment (1450 °C/100 h), the C14-NbCr_2_ Laves phase was still present in ZX8 and the Nb_ss_ was formed. The latter means that the Nb_ss_ was stable in the alloy ZX8 as it was in the heat treated alloy ZX4, but not in the heat treated alloy ZX6 (Table 1). These facts and those discussed above for the cast alloys would suggest that Al is the element that controls the stability of the Nb_ss_ in alloys with a high Sn content, and that Cr can “oppose” this. In other words, (i) in Nb-24Ti-18Si-5Sn silicide based alloys the additions of Al and Cr each at 5 at.% respectively hinder (alloy ZX6) and promote (ZX4) the stability of Nb_ss_, but the latter is stable when the Sn concentration in the alloy is lower at 2 at.% [13], and (ii) in Nb-24Ti-18Si silicide based alloys the vol.% of the Nb_ss_ can be controlled via the concentrations of Al, Cr, and Sn. 

The Nb_3_Sn was stable in all three alloys. The Si + Sn and Si + Sn + Al concentrations and the Si/Sn ratios in Nb_3_Sn in as cast and heat treated Nb-silicide based alloys with 5 at.% Sn, with/out Ti and without Hf are compared in Table 19. The data confirm that Al decreases the Si + Sn concentration and increases the Si/Sn ratio, and would suggest that the average Si + Sn + Al concentration in Nb_3_Sn is about 19.7 at.%. 

#### 5.2.4. Precipitation in αNb_5_Si_3_

In all three heat treated alloys there was evidence of fine precipitates inside the αNb_5_Si_3_ that exhibited contrast similar to that of the Nb_ss_ and Nb_3_Sn, particularly in the alloys ZX4 and ZX6. The X-ray maps for the latter indicated that these precipitates were Sn rich (Figure 1c,d and Figure 2d,e). Such fine precipitates in Nb_5_Si_3_ were not observed in the heat treated alloys NV9 and NV6 [22] and ZX3 [13]. The precipitation of second phase in αNb_5_Si_3_ with contrast similar to that of Nb_ss_ has been reported by our group in heat treated Al containing Nb-24Ti-18Si silicide based alloys without Sn e.g., [23,29] and in the Nb-21.1Si-8.3Ti-5.4Mo-4W-0.7Hf alloy [30]. The precipitation of Nb_ss_ in αNb_5_Si_3_ in the Nb-20Ti-18Si-4Hf-5Cr-3Al-1.5Sn alloy was studied in [11]. Sekido et al reported the precipitation of Nb_ss_ in βNb_5_Si_3_ and αNb_5_Si_3_ in Nb-xMo-36Si and Nb-*x*Mo-37.5Si (at.%, *x* = 0 to 10) alloys [31]. Precipitation was also observed in Ti rich Nb_5_Si_3_ in the heat treated Nb-24Ti-18Si-5Fe-5Sn alloy (NV8 in [32]) and in the Nb_5_Si_3_ in the heat treated Nb-24Ti-18Si-5Cr-5Fe-5Sn alloy (NV5 in [33]), where the precipitates were the Nb_3_Sn phase. The precipitation of a second phase in Nb_5_Si_3_ with contrast similar to that of Nb_ss_ or Nb_3_Sn was also observed in the low Sn content alloys ZX5 and ZX7 after heat treatment. The available evidence would thus suggest (i) that precipitation of a second phase in Nb_5_Si_3_ is not necessarily linked with the presence of Al or Sn in Nb-silicide based alloys, (ii) that the precipitates can be the A2 (bcc) Nb_ss_ or A15-Nb_3_X (X = Al, Si, Sn), and (iii) that Nb_ss_ precipitates can form in Nb_5_Si_3_, even in Sn containing alloys. Furthermore, the data for the alloys ZX3, ZX7 [13], ZX4, ZX6, and in [11] would suggest that whether Nb_ss_ or Nb_3_Sn precipitates in Nb_5_Si_3_ depends on the Sn concentration in the alloy.

### 5.3. Oxidation

#### 5.3.1. Oxidation at 800 °C

The starting (cast) microstructures of the three alloys were summarised in Table 1. The isothermal oxidation data was shown in the Figure 6a,c,e and the oxidation rate constants and weight gains of the alloys were summarised in Table 3. The alloys did not pest (Figure 5a,c,e). The WDS analysis data for the oxides in the scales of the alloys are given in Table 5 and Table 15. The oxides in the scales, the phases in the diffusion zone, and the bulk microstructures of the oxidised alloys are summarised in Table 20. The chemical compositions of the Nb rich and Nb and Si rich oxides formed in the scales of the three alloys are compared in Table 21 and the average chemical compositions of the Nb_ss_, Nb_5_Si_3_, and Nb_3_Sn in the bulk microstructures of the oxidised alloys, for which the data are given in Table 4, Table 6 and Table 8, are compared in Table 22. Note that, for the solid solution, there are only data for the alloy ZX4.

The TG data showed less breakaway oxidation for the alloys ZX4 and ZX6, compared with the alloy ZX8. Up to about 20.8 h the weight gain of the latter exhibited essentially the same behaviour as the alloy ZX4, but after that time the weight gain of the former (ZX8) accelerated relative to the latter, and after about 87.5 h it was equal to the weight gain of the alloy ZX6 and exceeded that over longer times. However, the data for the alloy ZX8 must be considered with care for the reasons discussed in the Section 4.3.1. The lowest weight gain was exhibited by the alloy ZX4 (Table 3). 

Considering the Nb rich oxide in the scales of the alloys ZX4, ZX6, and ZX8 compared with the lower Sn content alloys ZX3, ZX5, and ZX7 [13], the concentrations of Al and Cr in the oxide increased when the latter two elements were present in the alloy simultaneously (alloy ZX8) (Table 15). The concentrations of Sn and Cr decreased and Al increased in the Nb rich oxide when Al and Cr were present individually in the alloys compared with the low Sn content alloys ZX3, ZX5, and ZX7. In the “best” alloy at 800 °C (alloy ZX4) the Nb rich oxide had the lowest (Nb + Ti)/Si ratio (Table 21).

In the alloy ZX4, the Nb and Si rich oxide had the highest Si and Si + Sn, and the lowest Sn and (Nb + Ti)/Si of all the studied alloys (i.e., including the low Sn content alloys ZX3, ZX5, and ZX7). The Nb_ss_ was contaminated by oxygen in the bulk. Comparison with the data for the low Sn content alloys would suggest that the contamination of the solid solution in the bulk increased as the Sn concentration in the alloy increased. However, in the alloys with low and high Sn content, the Si/Sn ratio for the Nb_ss_ in the bulk was essentially the same, about 0.57. In the alloy ZX4, the Cr and Sn concentrations in the Nb_ss_ were the highest of all studied alloys (i.e., including the low Sn content alloys ZX3, ZX5 and ZX7) and the Nb_ss_ of this alloy also had the lowest Ti/(Al + Cr + Si + Sn) and Nb/Ti ratios and the highest Si + Sn and Ti + Si + Sn + Al + Cr concentrations. In the “worst” alloy at 800 °C (ZX5 [13]) the Nb_ss_ had the highest Ti/(Al + Cr + Si + Sn) ratio.

There was contamination by oxygen of the Nb_5_Si_3_ in the bulk of all the alloys after oxidation at 800 °C. The increase of Sn concentration in the alloy resulted in a slight increase in the Sn concentration in the Nb_5_Si_3_, but did not have any strong effects on the composition of Nb_5_Si_3_ in the bulk. The Nb_3_Sn in the bulk of the alloys was also contaminated by oxygen, but the concentration of the latter was the same (about 6 at.%, Table 22) regardless of whether the Al and Cr were present individually or simultaneously in the alloys. In the “best” alloy at 800 °C (alloy ZX4) the Nb_3_Sn had the highest Si and Sn concentrations. 

#### 5.3.2. Oxidation at 1200 °C

The starting microstructures of the three alloys were summarised in Table 1. The isothermal oxidation data was shown in the Figure 6b,d,f and the oxidation kinetics data and weight gains of the alloys were summarised in Table 3. The scales that formed on the alloys spalled off (Figure 5b,d,f). The WDS analysis data for the oxides in the scales of the alloys is given in Table 15. The thickness of the scales and the Sn rich area, the oxides in the scales, the phases in the Sn rich area, and the bulk microstructures of the oxidised alloys are summarised in Table 23. The chemical compositions of the Nb, Nb and Si, and Ti rich oxides that formed in the scales of the alloys ZX6 and ZX8 are compared in Table 24. Note that there are no data for the alloy ZX4. The average chemical compositions of the Nb_ss_, Nb_5_Si_3_, and Nb_3_Sn in the bulk microstructures of the oxidised alloys, for which the data are given in Table 10, Table 12 and Table 14, are compared in Table 25. Note that for the solid solution, there are data only for the alloy ZX4. 

The alloys started oxidation at 1200 °C with only the alloy ZX8 not having the Nb_ss_ in its micro-structure. The contamination of the latter alloy by oxygen did not stabilise the Nb_ss_ during oxidation at 1200 °C. In the alloy ZX6, the Nb_ss_ was not stable after the heat treatment at 1500 °C (see Section 5.2) and the Nb_ss_ was not observed in the oxidised alloy ZX6. The Nb_ss_ was not present in both the oxidised alloys ZX6 and ZX8, of which the former gained less weight at 1200 °C.

The TG data (Figure 6 and Table 3) suggested break-away oxidation only for the alloy ZX4 even though the latter had almost the same linear oxidation rate constant with the alloy ZX8. These two alloys contained the Laves phase in their starting microstructures and, after oxidation at 1200 °C, had gained the same weight in the early stages of oxidation (up to about 8.3 h) and after about 100 h. However, between these times, the weight gain of the former increased, owing to the breakaway oxidation, which would suggest that the simultaneous presence of Al and Cr in the latter alloy improved the mechanical behaviour of the scale when the Sn content in the alloy had increased. This phenomenon must be attributed to Al, as the weight gains of the two Al containing alloys, ZX5 and ZX6, were essentially not significantly different, but the oxidation of the alloy ZX6 (higher Sn content) was linear throughout while that of ZX5 was initially parabolic followed by linear behaviour with a linear rate constant slightly lower than ZX6. This would suggest that with Al in the alloy, the 2 at.% Sn addition is good enough for oxidation at 1200 °C. However, as we discussed in the previous section, the higher Sn content of ZX6 compared to ZX5 was beneficial for pest oxidation. 

Figure 11 shows that three oxides were present in the scale, namely Nb rich, Nb and Si rich, and Ti rich oxides. As the Sn content of the alloys increased, Ti rich oxide formed in the scale of the alloy ZX6 but was not observed in the scale of the alloy ZX5 [13]. The increase in Sn content in the alloys was accompanied by thicker Sn rich areas at the scale/substrate interface, which is consistent with the surface segregation of a solute element being dependent on the bulk concentration [13]. However, in all three alloys, the thickness of this area was essentially the same (≈50 μm, Table 23) but varied between the low Sn content alloys [13]. In the Nb–Sn binary, the NbSn_2_ compound is not stable above 830 °C [21]. The same Sn rich intermetallic phases were formed in the Sn rich areas of the alloys with the exception of NbSn_2_ that was formed only in the alloy ZX4 (Table 23), which gained slightly more weight than the other two alloys (Table 3). This observation and the data for the low Sn content alloys, where NbSn_2_ was not formed in the Sn rich area of the alloy ZX5 but was formed in the alloy ZX7 [13], would suggest that the formation of NbSn_2_ in the substrate microstructure below the scale/substrate interface was detrimental to oxidation at 1200 °C.

The Nb rich oxide that formed in the scales of the alloys essentially did not contain Cr, Si, and Sn (Table 15) and, compared with the same oxide type that formed at 800 °C, it had the same Nb + Ti content but higher Nb/Ti ratio (Table 21 and Table 24). The concentrations of Al, Cr, and Sn in the Nb and Si rich oxide that formed in the scale of the alloys were negligible (Table 15) with similar Nb + Ti and Nb/Ti ratios (Table 24). The concentrations of the same elements in the Nb and Si rich oxides that formed at 800 °C were very low (Table 21). In the Ti rich oxide that was observed only at 1200 °C, the concentrations of Al and Cr were low but not zero, and comparable with those in the Nb rich oxide that formed at 800 °C. The Nb + Ti content of the Ti rich oxide was between those of the other two oxides formed at 1200 °C and, like the Nb rich oxide, it was essentially free of Si and Sn. The Nb/Ti ratio did not change (Table 24).

The data for all three alloys would suggest (i) that with the higher Sn content, the presence of C14-NbCr_2_ Laves phase is not an essential requirement for achieving the “best” oxidation behaviour at 1200 °C and (ii) that with only Sn as the extra alloying addition (meaning in addition to Al, Cr, and Ti) that improves the oxidation resistance, the spallation of the scale could not be avoided, even though the synergy of Al with Sn seemed to improve the adhesion of the scale on the alloy ZX6 (Figure 5d). In the latter, compared with the alloy ZX8, (a) the Nb rich oxide was richer in Al (Table 15), had lower (Nb + Ti)/(Si + Al + Sn) and (Nb + Ti)/(Al + Cr + Sn) ratios and higher Nb/Ti ratio (Table 24), (b) the Ti rich oxide was richer in Al and Ti (Table 15) and also had a lower (Nb + Ti)/(Si + Al + Sn) ratio and similar Nb/Ti ratio (Table 24), and (c) the Nb and Si rich oxide was poorer in Si (Table 15), had a lower (Nb + Ti)/(Si + Al + Sn) ratio and similar Nb/Ti ratio (Table 24). In other words, in the scale with the “better” adhesion, the (Nb + Ti)/(Si + Al + Sn) ratio was lower. On the substrate, owing to the addition of Al in ZX6, the substitution of Sn and Si by Al in Nb_3_Sn and Nb_5_Sn_2_Si would be expected to increase the Poisson’s ratio ν and to lower the Young’s modulus E and the ratio G/B (G = shear modulus, B = bulk modulus) in both compounds, making them “more ductile” (data for A15-Nb_3_X in [34] and for TM_5_Sn_2_X (TM = Nb, Ti, X = Al, Si) in [35]). The NbSn_2_ was not formed in ZX6 (was present only in the Sn rich area of ZX4). The latter compound has lower ν and higher G/B [36] than both Nb_3_X (X = Al, Sn) and TM_5_Sn_2_X and is thus expected to be “less ductile”.

The solubility of oxygen in the Nb_ss_ in the bulk of the alloy ZX4 decreased with the increase in the Sn content in the alloy. Compared with the Si content of the Nb_ss_ in the heat treated alloy ZX4, the Si content in the Nb_ss_ in the bulk of the alloy after oxidation at 1200 °C was high (Table 25) and out of step with other data from our research group. It is therefore suggested that it is highly likely that the Si concentration in Table 25 is wrong, probably due to analysis error. If we were to accept this, the parameters Si + Sn, Si/Sn and Nb/Ti in the Nb_ss_ did not change significantly with the increase of the Sn content of the alloy. If we were not to accept the above proposition, then only the ratio Nb/Ti did not change significantly with ab increase of the Sn content of the alloy. The solubility of oxygen and the Si + Sn and Si + Sn + Al concentrations and Nb/Ti ratio in the Nb_5_Si_3_ in the bulk of the alloys at 1200 °C did not change as the Sn content of the alloys increased but the Sn concentration in the silicide increased. In Nb_3_Sn, the oxygen solubility was the same as in the Nb_ss_ of the alloy ZX4 (Table 25) and the Nb_3_Sn at 800 °C (Table 22) and did not change with increasing Sn in the alloys, but the Sn concentration increased. At 1200 °C the Nb/Ti ratio did not change significantly compared with 800 °C (Table 22 and Table 25) and with increasing Sn content in the alloy.

The parameters δ (related to atomic size), Δχ (related to electronegativity), and number of valence electrons per atom filled into the valence band (VEC) describe well the alloying behaviour (a) of Nb-silicide based alloys [15] and (b) of the most important phases in their microstructures [16,17,18,19]. Also, there are relationships between the properties of these alloys (creep, weight gain in isothermal oxidation) and their phases and the alloy or phase parameters δ, Δχ and VEC [17,18,19]. These relationships are used in the alloy design methodology NICE [14] to design new Nb-silicide based alloys. NICE is also used to design complex concentrated alloys (CCAs) or high entropy alloys [25]. NICE shows that, for oxidation resistance, the trends of VEC and δ are opposite and that alloy design should aim to decrease the former and increase the latter. Recently, this was demonstrated in [37]. 

**Figure 12:**
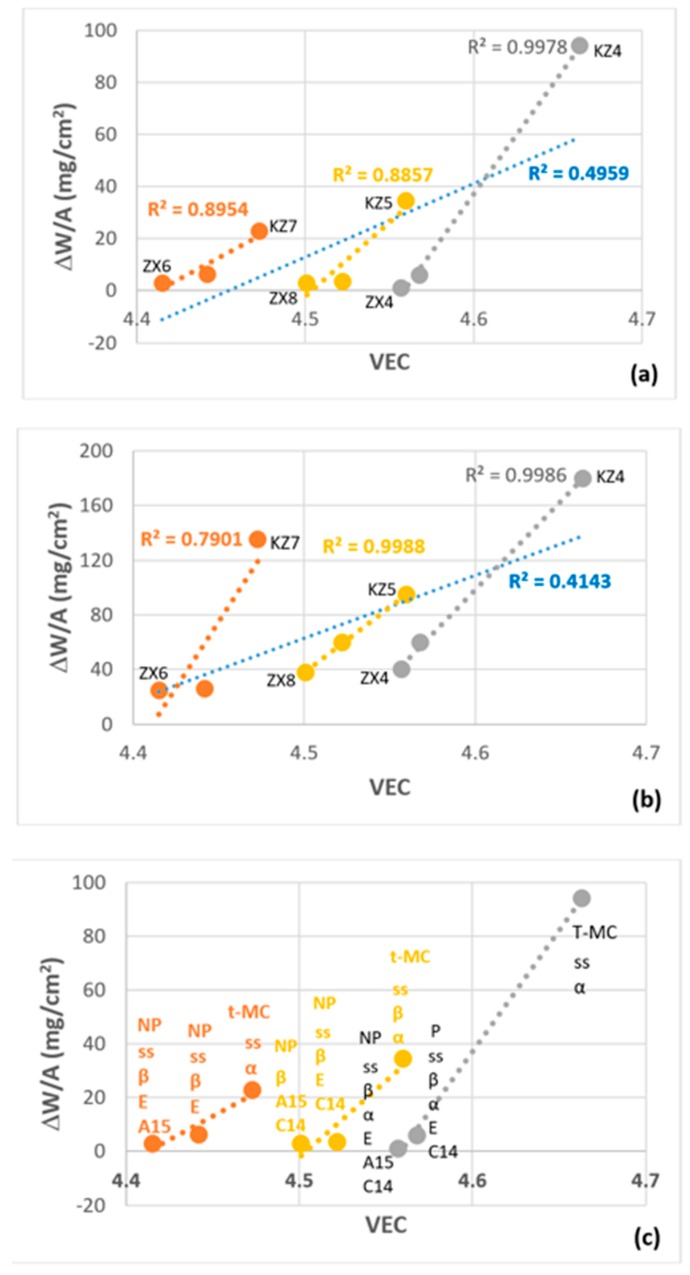
(**a**,**b**) weight gains per unit area versus VEC respectively for 800 °C and 1200 °C and (**c**) summary of phases present in the microstructure of the specimens of the alloys used for the isothermal oxidation experiments in pest regime.

Figure 12a,b shows plots of weight gain per unit area data versus the parameter VEC at 800 and 1200 °C. The latter was calculated using the actual compositions of the alloys. In this figure, data are included for the low and high Sn content alloys, respectively ZX3, ZX5, ZX7 [13] and ZX4, ZX6, ZX8, as well as for the equivalent alloys without Sn, namely the alloys KZ4, KZ7, and KZ5 [23,38]. The trend of all data at each temperature is shown by the blue dotted line with a low R^2^ value. For each temperature, the linear fit of the data is significantly improved (higher R^2^ value) when the alloys are separated in groups according to the synergy of Sn with Al or Cr, or with both of these elements. All the data and the data in each group show that the weight gain per unit area in isothermal oxidation at 800 or 1200 °C decreases as the parameter VEC decreases, in agreement with NICE [14]. For the same alloys, the weight gain per unit area in isothermal oxidation at 800 or 1200 °C decreases as the parameter δ increases (figure not shown), in agreement with NICE. Similar trends were observed for equivalent Nb-24Ti-18Si based alloys with Ge instead of Sn [37].

The phases present in the starting microstructures of the oxidation specimens are included in the Figure 12c, which is the same as Figure 12a without the linear fit lines, the R^2^ values and the alloy codes. The same phases in each alloy would be shown if, instead, the Figure 12b was used. The weight gains and VEC values decreased when the C14-NbCr_2_ Laves phase was present (ZX3 versus KZ4, and ZX7 versus KZ5) and decreased even further when the Nb_3_Sn was present with the Laves phase (ZX3 versus ZX4, and ZX7 versus ZX8) or without the Laves phase (KZ7 versus ZX5 versus ZX6). At both temperatures, the strongest effect of Sn addition was observed for the alloys where Sn was in synergy only with Cr (KZ4 versus ZX3 and ZX4). Pest oxidation was suppressed in Cr containing alloys only when the Sn concentration increased to 5 at.% (ZX3 versus ZX4). In the Al, and Al and Cr containing alloys, the suppression of pesting was achieved with 2 at.% Sn with/out the presence of Laves phase (ZX5 and ZX7). With the increase of Sn concentration, which stabilised the Nb_3_Sn, pesting was also suppressed with/out the Laves phase in the microstructure (ZX6 and ZX8). Note (a) that the alloy ZX6, which occupies the left hand side, bottom part of Figure 12a,b, gained less weight at 1200 °C, where the adhesion of its scale was “better” than that of the other alloys, (b) that the weight gains of the alloys with 5 at.% Sn (ZX4, ZX6, ZX8) were not very different at both temperatures, but their VEC values are (the same is the case with the parameter δ), and (c) that with Al in the alloy, the 2 at.% Sn addition should most probably be good enough for oxidation at 1200 °C. 

#### 5.3.3. Sn Rich Areas

After isothermal oxidation at 800 and 1200 °C, all three alloys formed Sn rich areas in the substrate below the scale/substrate interface, the thickness and continuity of which increased with the oxidation temperature (and with the Sn concentration in the alloys when the data is compared with the low Sn content alloys ZX3, ZX5 and ZX7). Owing to the characteristic features of the Sn rich areas, the analysis of Sn rich intermetallics was possible mainly in the cross sections of the alloys that were oxidised at 1200 °C. In the Sn rich areas, different Sn rich intermetallics were formed, namely the NbSn_2_, Nb_5_Sn_2_Si, Nb_3_Sn, the former two with Ti, Al, and Cr and the latter with Ti, Cr, Al, and Si in their composition. These intermetallics were contaminated by oxygen (Table 9, Table 11 and Table 13). The enrichment of the substrate at its interface with the scale with Sn was discussed in [13].

Figure 8b suggests that the existing Nb_3_Sn in the microstructure of ZX4 played a role in the enrichment of the diffusion zone below the scale with Sn. This figure shows that a transformation of the Nb_3_Sn occurred at a planar interface (indicated by a dashed line) with lamellar features. In the Nb–Sn binary [21], the Nb_3_Sn transforms to Nb_ss_ + Nb_6_Sn_5_ via a eutectoid transformation and the Nb_6_Sn_5_ transforms to Nb_ss_ + NbSn_2_ also via a eutectoid transformation. It is suggested that, as the surface regions became enriched with Sn, the Nb_3_Sn formed, and the composition of the Nb_3_Sn (newly formed and existing) moved close to the composition for the first of the two eutectoid reactions mentioned above, such transformations leading to the microstructure seen in Figure 8b.

In the Sn rich area in the alloy ZX6 the Nb_5_Si_3_ was observed with Si + Al + Sn = 37.4 at.%, (Si + Al + Sn)/O_2_ = 9.1 and 8 < Ti < 16 at.%, compared with 36 at.% and 8.1 for the former two parameters and Ti = 15.6 at.% in the bulk, which would suggest that the Ti of some Nb_5_Si_3_ grains near the scale had been consumed to form the Ti rich oxide. In the Sn rich area, the Nb_3_Sn had Si + Al + Sn = 24.49 at.%, Si/Sn = 0.04 and (Si + Al + Sn)/O_2_ = 3.4, similar to the values of these parameters for the same phase in the alloy ZX5 at 1200 °C. However, in the Sn rich area of the alloy ZX6, there was Nb_3_Sn with composition similar to the Nb_3_Sn in the bulk. This Nb_3_Sn (i.e., the one in the Sn rich area) had Si + Al + Sn = 17.6 at.%, Si/Sn = 0.37 and (Si + Al + Sn)/O_2_ = 2.9 compared with 16.6 at.%, 0.36 and 2.98 of the same parameters in the bulk. In other words, in the Sn rich area, in the alloy ZX6 there were “existing” Nb_3_Sn grains and “newly formed” Nb_3_Sn from the enrichment in Sn, which was also responsible for the formation of Nb_5_Sn_2_Si. The Nb_5_Sn_2_Si was observed in the Sn rich area in ZX6 with (Si + Al + Sn)/O_2_ = 5.4, the same as in ZX5 at 1200 °C. 

In the Figure 9 the analyses 65, 68 show Sn rich areas form from Nb_3_Sn (Table 11), in agreement with ZX6 at 800 °C (Figure 7d) and the data for ZX4 (Figure 8b). Details of the microstructures indicated by circles in the Figure 9a,b are shown in Figure 9c,d. Similar features were observed in the Sn rich area formed in the alloy ZX3 at 1200 °C. The microstructures in Figure 9c,d suggest recrystallization in the Sn rich area. If the latter was indeed the case, then it is worth considering possible source(s) for the strain energy that drove recrystallization.

In (a) and (b) the linear fit of all data is shown by the blue dotted lines with lowest R^2^ values. In (c) ss = Nb_ss_, β = βNb_5_Si_3_, α = αNb_5_Si_3_, E = eutectic, C14=C14-NbCr_2_ Laves phase, A15 = Nb_3_Sn, T-MS = thick “maltese cross” scale, t-MC = thin scale that could develop to “maltese cross”, P = pest, NP = no pest. In (a) to (c) colours as follows: orange; alloys with Al (KZ7 [23], ZX5 [13], ZX6), grey; alloys with Cr (KZ4 [23], ZX3 [13], ZX4), gold; alloys with Al and Cr (KZ5 [23], ZX7 [13], ZX8). The nominal compositions of the alloys KZ4, KZ5 and KZ7 respectively were Nb-24Ti-18Si-5Cr, Nb-24Ti-18Si-5Al-5Cr and Nb-24Ti-18Si-5Al. (Note that the oxidation specimens of the alloys KZ4, KZ5 and KZ7 were selected from heat treated alloys).

As we discussed above, the Sn rich areas were observed in the oxidised alloys. During the oxidation of a specimen, the oxidation of the different phases in the microstructure of the substrate depended on the chemistry and crystal structure of phases. The diffusion of oxygen is expected to be easier via the Nb_ss_ owing to the inherent high solid solubility of oxygen in the latter [21] and reactive solute elements like Ti and Al. The inward diffusion of oxygen was accompanied by the enrichment of the surface area of the substrate by Sn, and the formation of Sn rich intermetallics. 

The formation of oxides, the enrichment of the surface areas with Sn prior to the formation of Sn rich intermetallics, the contamination of existing phases like Nb_ss_, Nb_5_Si_3_, and Nb_3_Sn, and the formation of Sn rich intermetallics must have been accompanied with increased defect density and internal stress levels arising from differences in the density of phases, their coefficients of thermal expansion, and moduli of elasticity. For example, the moduli of elasticity of Nb, βNb_5_Si_3_, αNb_5_Si_3_ and Nb_3_Sn respectively are 105, 269, 291 [39], and 173 GPa [36], but for Nb_3_Sn, the values of 127 GPa [40], 195 GPa [41], and 132 GPa [42] have been reported, the latter for 273 °C. Furthermore, the modulus of elasticity of Nb_6_Sn_5_ is 144 GPa [36] and of Nb_3_Al (which has the A15 structure like the Nb_3_Sn) is 164 GPa [34] or 193 GPa [41]. The CTE (coefficient of thermal expansion) of Nb is 7.6 × 10^−6^ K^−1^ and of Nb_3_Sn is 10.3 × 10^−6^ K^−1^ [43]. The βNb_5_Si_3_ and αNb_5_Si_3_ exhibit anisotropy in CTE and the ratio of CTE values along the a and c axes of their lattices (i.e., the CTE anisotropy ratio) is different for each phase [44]. All the aforementioned values are for the unalloyed phases and are expected to change as they become alloyed [44]. 

To demonstrate the dependence of stresses arising in the Sn rich area from differences in the E and CTE values of phases, the stress is estimated as σ = E_SRA_E_sub_ ΔT(α_sub_ − α_SRA_)[E_sub_ + 2E_SRA_ (d_SRA_/d_sub_)]^−1^ [45] where E_i_ and α_i_ respectively are moduli of elasticity and coefficient of thermal expansion, ΔT is temperature difference and d_i_ is thickness [i = SRA (Sn rich area) or Sub (substrate)]. If we consider the data given above for Nb and Nb_3_Sn, with the former as the substrate and the latter the phase formed in the SRA and take d_SRA_ = 10 μm and d_sub_ = 100 μm, then the calculated stress for ΔT = 1000 K is |σ| = 276 MPa for the lowest reported modulus of elasticity of Nb_3_Sn (127 GPa) and |σ| = 384 MPa for the highest reported value (195 GPa). For d_SRA_ = 50 μm and d_sub_ =100 μm, the corresponding stresses for ΔT = 1000 K are |σ| = 155 MPa and 184 MPa. For d_SRA_ = 1 μm and d_sub_ = 100 μm, the corresponding stresses for ΔT = 1000 K are |σ|= 343 MPa and 508 MPa. The estimated values of σ show that the stress is very high at the early stages of SRA formation and decreases as d_SRA_ increases and, for the same d_SRA_, increases with the modulus of elasticity of the substrate. Given the high homologous temperatures of the oxidation experiments, recovery processes, and therefore a gradual reduction in defect density and stresses, should have occurred before recrystallization set-in in parts of the Sn rich area in an oxidised alloy driven by a reduction in strain energy that depended on the substrate phase(s) as well as the thickness and the type(s) of SRA phase(s) formed.

In the alloy ZX8 after oxidation at 800 °C, a two phase Nb_ss_ + Nb_5_Si_3_ eutectic like microstructure, which was contaminated by oxygen, was observed (Figure 7f), even though the Nb_ss_ and a Nb_ss_ + Nb_5_Si_3_ eutectic were not present in the cast alloy. The Si + Al + Sn concentration of the Nb_ss_ + Nb_5_Si_3_ microstructure was about 17 at.%, close to that of the Nb_ss_ + Nb_5_Si_3_ lamellar microstructure in the bulk of the oxidised alloy ZX6 (about 18 at.%) (see Figure 7c). The formation of the Nb_ss_ + Nb_5_Si_3_ eutectic like microstructure in ZX8 at 800 °C was attributed to changes in phase equilibria due to contamination by oxygen. The same phases were observed in the Sn rich area of the alloy ZX8 at 1200 °C as in the alloy ZX7 with the exception of NbSn_2_, and Sn rich areas were also formed in the Nb_5_Si_3_, as was the case in ZX7 [13]. In the bulk microstructure, the Nb_5_Si_3_, Nb_3_Sn, and Laves phase were present. Thus, contrary to the formation of the Nb_ss_ and Nb_ss_ + Nb_5_Si_3_ lamellar structure in the oxidised alloy ZX8 at 800 °C, the Nb_ss_ was not stabilised in the microstructure at 1200 °C, even though it was present in the heat treated microstructure, meaning that, in the new phase equilibria with oxygen at 1200 °C, the solid solution was not stable.

## 6. Concluding Remarks

This research was motivated by the need to understand how Sn improves the oxidation resistance of Nb-silicide based alloys. We discussed the selection of the Sn concentration in Nb-silicide based alloys of the Nb-Ti-Si-Al-Cr-Sn system and presented the results of a systematic study of three alloys of nominal compositions, namely Nb-24Ti-18Si-5Cr-5Sn (ZX4), Nb-24Ti-18Si-5Al-5Sn (ZX6), and Nb-24Ti-18Si-5Al-5Cr-5Sn (ZX8).

- There was macrosegregation in all three alloys that was most severe in ZX8.
- The Nb_ss_ was not stable in ZX6, the Nb_3_Sn was stable in all three alloys and the Nb_ss_ and the C14-NbCr_2_ Laves phase were stable in ZX4 and ZX8.
- In all three alloys, the primary βNb_5_Si_3_ transformed completely to αNb_5_Si_3_ after heat treatment.
- The 5 at.% Sn addition suppressed pest oxidation at 800 °C, but not the spallation of scales at 1200 °C.
- At 800 and 1200 °C, a Sn-rich area developed below the scale in the substrate/scale interface where Nb_3_Sn, Nb_5_Sn_2_Si and NbSn_2_ compounds were formed. The latter two compounds were most noticeable at 1200 °C. The Sn-rich area was thicker at 1200 °C and was contaminated by oxygen at both temperatures.
- The bulk of all three alloys was contaminated by oxygen, and the contamination of the Nb_ss_ was more severe.
- Nb-rich, Ti-rich, and Nb and Si-rich oxides formed in the scales. The scale that formed on the alloy ZX6 at 1200 °C adhered better on the substrate compared with the other two alloys.
- Improvement of oxidation resistance at both temperatures was accompanied by a decrease and increase, respectively, of the alloy parameters VEC and δ, in agreement with the alloy design methodology NICE.

Considering the questions that were presented in the introduction of the paper:

- a higher Sn concentration seems to be essential for suppressing the pest oxidation of Nb-24Ti-18Si based alloys with Cr and no Al additions, but not for alloys where Al and Cr are in synergy with Sn,
- the stability of Nb_3_Sn in the alloy is “assured” with 5 at.% Sn addition and does improve oxidation resistance with/out the presence of the Laves phase and
- the synergy of Sn with Al, both of which form A15 compounds and Al substitutes Si in Nb_5_Sn_2_Al, gives the “best” oxidation behaviour with improved scale adhesion at the high temperature.

## Figures and Tables

**Table 1:** Average compositions (at.%) of the cast and heat treated alloys ZX4, ZX6 and ZX8 and summary of the phases confirmed by XRD and microanalysis.

Alloy	As Cast		Heat Treated	
	Composition	Phases	Composition	1500 °C/phases
ZX4	Nb-25.4Ti-18.8Si-4.9Cr-5Sn	αNb_5_Si_3_, βNb_5_Si_3_, Nb_ss_, Nb_3_Sn, C14-NbCr_2_ Laves, (Nb_ss_ + Nb_5_Si_3_)_eutectic_, (Nb_ss_ + NbCr_2_ + Nb_5_Si_3_)_eutectic_	Nb-26Ti-20Si-4.3Cr-5.1Sn	Nb_ss_, Nb_3_Sn, αNb_5_Si_3_, C14-NbCr_2_ Laves
ZX6	Nb-25.2Ti-18.5Si-4.9Al-5Sn	βNb_5_Si_3_, Nb_ss_, Nb_3_Sn, (Nb_ss_ + Nb_5_Si_3_)_eutectic_	Nb-23.9Ti-19.3Si-4.5Al-4.4Sn	αNb_5_Si_3_, Nb_3_Sn
				1450 °C/phases
ZX8	Nb-23.9Ti-17.2Si-5.4Cr-4.8Al-4.6Sn	βNb_5_Si_3_, Nb_3_Sn, C14-NbCr_2_ Laves	Nb-21.1Ti-18.6Si-5.1Cr-4Al-4.6Sn	αNb_5_Si_3_, Nb_3_Sn, Nb_ss_, C14-NbCr_2_ Laves

**Table 2:** Macrosegregation (at.%) of elements in the as cast alloys ZX4, ZX6 and ZX8.

Alloy	MACi (i = Al,Cr,Si,Ti)			
	Al	Cr	Si	Ti
ZX4		3.6	7.3	6.6
ZX6			5.5	7.3
ZX8	1.9	4.7	10	9.7

**Table 3:** Weight gain per unit area ΔW/A (mg cm^−2^) and linear k_l_ (g cm^−2^s^−1^) and parabolic k_p_ (g^2^ cm^−4^ s^−1^) oxidation rate constants of the alloys ZX4, ZX6 and ZX8 for isothermal oxidation at 800 and 1200 °C.

T (°C)	Alloy					
	ZX4		ZX6		ZX8	
800	K_p_ = 4.7 × 10^−12^	ΔW/A ≈ 1	K_p_ = 2.2 × 10^−11^ (t ≤ 25 h) K_l_ = 6.4 × 10^−9^ (t > 25 h)	ΔW/A ≈ 3	K_p_ = 2.8 × 10^−12^ (t ≤ 22 h K_l_ = 1 × 10^−8^ (t > 22 h)	ΔW/A ≈ 3
1200	K_p_ = 1.65 × 10^−9^ (t ≤ 16.7 h) K_l_ = 1 × 10^−7^ (t > 16.7 h)	ΔW/A ≈ 40	K_l_ = 7.3 10^−8^	ΔW/A ≈ 25	K_l_ = 1.1 × 10^−7^	ΔW/A ≈ 38

**Table 4:** WDS analysis data (at.%) of the phases in the bulk of the oxidised alloy ZX4 at 800 °C.

Phase	Nb	Ti	Si	Cr	Sn	O
	43.6 ± 0.14	15.6 ± 0.39	34 ± 0.67	1.1 ± 0.08	1.5 ± 0.46	4.2 ± 0.62
Nb_5_Si_3_	43.4–43.8	15.1–16.3	33–34.8	0.9–1.2	0.6–1.9	3.3–4.7
	55 ± 0.84	20.5 ± 0.92	5.9 ± 0.20	2.9 ± 0.55	9.4 ± 0.64	6.3 ± 0.55
Nb_3_Sn	53.9–56.2	19.5–21.9	5.7–6.3	2.4–3.8	8.3–10.1	5.8–7.1
	46.7 ± 1.23	29.4 ± 1.07	2.7 ± 0.76	9.2 ± 0.67	4.8 ± 0.38	7.2 ± 0.81
Nb_ss_	45.8–48.8	28–31	1.7–3.7	8.6–10.3	4.5–5.5	5.5–8

**Table 5:** WDS analysis data (at.%) of phases in the scale and the Sn rich area below it in the alloys ZX6 at 800 °C. For the numbers of analyses, see Figure 7g.

Sn Rich Area	Nb	Ti	Si	Al	Sn	O
Spot 10	33.2	13.2	5.6	4	16.7	27.3
Spot 11	30.6	14.2	3.9	3.7	16.4	31.2
Spot 14	40.3	8.8	4.1	4.3	21.9	20.6
**Scale**						
Spot 21	16.1	5.8	11.5	0.7	0.3	65.6
Spot 22	14.2	9.4	7.3	1.6	0.7	66.8
Spot 23	18.4	9.7	1.5	2.5	0.8	67.1
Spot 24	16.6	11.9	1.2	2.1	0.3	67.9
**Nb_3_Sn**						
Spot 17	55	20.7	5.1	4.3	9.1	5.8
Spot 18	51.1	23.3	5.1	4.9	7.8	7.8
Spot 19	54.3	21.9	5.2	4.5	8.8	5.3
**Nb_5_Si_3_**						
Spot 13	42.1	18	32.4	3.1	2.3	2.1
Spot 15	43.8	15.8	34	2.3	1.9	2.2
Spot 16	43.4	15.7	34.5	1.9	1.7	2.8
**Prior eutectic**						
Spot 25	35.5	31.8	10.9	4.1	3.1	14.6
Spot 26	34.4	31.2	9.2	4.1	3.4	17.7

**Table 6:** WDS analysis data (at.%) of the phases in the bulk of the alloy ZX6 at 800 °C.

Phase	Nb	Ti	Si	Al	Sn	O
Nb_3_Sn	52.8	22.7	4.9	4.5	8.2	6.9
Nb_5_Si_3_	43.3	16.4	33.6	2.4	1.9	2.4
Prior eutectic	40.9	35.8	10.1	4.6	3.6	5

**Table 7:** WDS analysis data (at.%) of the phases in the diffusion zone of the alloy ZX8 at 800 °C.

Phase	Nb	Ti	Si	Cr	Al	Sn	O
Nb_5_Si_3_	44.6	15.7	28.1	1.2	4.7	2.1	3.6
Nb_3_Sn	52	19.5	3.2	3.8	3.6	8.9	9

**Table 8:** WDS analysis data (at.%) of the phases in the bulk of the alloy ZX8 at 800 °C.

Phase	Nb	Ti	Si	Cr	Al	Sn	O
Nb_5_Si_3_	40.9	19.8	32.3	1.2	0.9	1	3.9
Nb_3_Sn	54.5	19.6	2.4	3.8	5.2	8.3	6.2
Nb_ss_ + Nb_5_Si_3_	37.3	29.5	7.7	8.2	6.4	3	7.9

**Table 9:** WDS analysis data (at.%) for the Sn rich area of the alloy ZX4 at 1200 °C.

Phase	Nb	Ti	Si	Cr	Sn	O
NbSn_2_	31	1.3	0.2	0.4	62.7	4.4
Nb_3_Sn	57.4	4.8	2.4	2.5	27.5	5.4
Nb_5_Sn_2_Si	47	10.2	11.9	2.1	23.8	5

**Table 10:** WDS analysis data (at.%) of the phases in the bulk of ZX4 at 1200 °C.

Phase	Nb	Ti	Si	Cr	Sn	O
	43.6 ± 0.36	15.4 ± 0.28	34.9 ± 0.48	0.9 ± 0.11	1.6 ± 0.09	3.6 ± 0.45
Nb_5_Si_3_	43.3–44.2	15.2–15.8	34.1–35.2	0.7–1	1.5–1.7	3.1–4
	48.8 ± 0.31	26.4 ± 0.37	2.9 ± 0.28	3.8 ± 0.22	12.4 ± 0.49	5.7 ± 0.40
Nb_3_Sn	48.6–49.4	25.8–26.8	2.7–3.4	3.5–4.1	11.6–12.7	5.2–6.2
	54.8 ± 0.46	26.4 ± 0.70	2.9 ± 1.65	6.4 ± 0.24	3.1 ± 0.17	6.4 ± 0.08
Nb_ss_	54.2–55.2	25.7–27.1	1.3–4.6	6.2–6.7	3–3.3	6.3–6.5

**Table 11:** WDS analysis data (at.%) of the phases in the Sn rich area of the alloy ZX6 at 1200 °C. For the numbers of spot analyses, see Figure 9.

Phase	Nb	Ti	Si	Al	Sn	O
**Nb_5_Si_3_**						
Spot 59	48.4	9.5	30.5	0.3	6.7	4.6
Spot 61	42.7	16	32.9	1.9	3.1	3.4
Spot 62	50.3	8.2	30.9	0.2	6.2	4.2
Spot 64	43.1	15.8	33.2	1.9	1.8	4.2
**Nb_5_Sn_2_Si**						
Spot 60	46.8	10.9	13.6	0.6	21.2	6.9
Spot 63	50.5	8.9	10.9	1.2	22.8	5.7
Spot 67	45.1	13.5	8.4	1.8	24.4	6.8
**Nb_3_Sn**						
Spot 65	54.2	21.5	3.3	5.4	8.6	7
Spot 66	56.3	11.9	1	1.7	21.9	7.2
Spot 68	55.7	21.5	3	6.1	8.7	5

**Table 12:** WDS analysis data (at.%) of phases in the bulk of the alloy ZX6 at 1200 °C.

Phase	Nb	Ti	Si	Al	Sn	O
	43.1	15.6	33.9	1.5	1.4	4.5
Nb_5_Si_3_	42.7–43.5	15.4–15.7	33.7–34.2	1.5–1.6	1.4–1.4	3.7–5.2
	54.2	23.5	3.1	5	8.6	5.6
Nb_3_Sn	53.3–55.3	22.8–24.2	2.7–3.2	4.5–5.4	8.2–8.9	4.9–7

**Table 13:** WDS analysis data (at.%) of the diffusion zone of the alloy ZX8 at 1200 °C. For the numbers of spot analyses, see Figure 10b.

Analysis	Nb	Ti	Si	Cr	Al	Sn	O
Spot 10	44.1	11.7	8.7	3	1.5	25.1	5.9
Spot 11	46	13.4	29.8	0.6	1.1	4.4	4.7
Spot 12	35.7	14.2	1.4	3.4	-	40.4	4.9
Spot 13	42.7	19.1	30.1	0.9	1.6	1.1	4.5
Spot 14	49.2	21.9	1.3	4.7	7.8	9.3	5.8

**Table 14:** WDS analysis data (at.%) of phases in the bulk of the alloy ZX8 at 1200 °C.

Phase	Nb	Ti	Si	Cr	Al	Sn	O
Nb_5_Si_3_	42	19	30.2	0.7	2.8	1.1	4.2
Nb_3_Sn	51.8	21.8	1.5	4.3	6.4	8.2	6

**Table 15:** WDS analysis data (at.%) of the oxides formed on the alloys at 800 and 1200 °C.

Alloy	Phase	Nb	Ti	Si	Cr	Al	Sn	O
		**800 °C**						
ZX4	Nb rich oxide	18.4	11	1.6	3		0.1	65.9
	Nb-Si rich oxide	14.2	5.8	13.3	0.5		0.2	66
								
ZX8	Nb rich oxide	19.6	8.8	0.6	4.7	1.5	0.2	64.6
	Nb-Si rich oxide	15.3	6.9	10.7	0.7	1.6	0.2	64.6
		**1200 °C**						
ZX6	Nb rich oxide	24.3	7.8	-		1.3	-	66.6
	Ti rich oxide	6.7	21.1	0.1		5.5	-	66.6
	Nb-Si rich oxide	14.7	5.6	13.6		0.5	-	65.6
								
ZX8	Nb rich oxide	20.6	7.7	0.1	0.2	0.2	-	71.2
	Ti rich oxide	7.1	17.6	0.2	3.5	1.9	0.4	69.3
	Nb-Si rich oxide	14.4	5.4	8.8	0.5	0.5	-	70.4

**Table 16:**
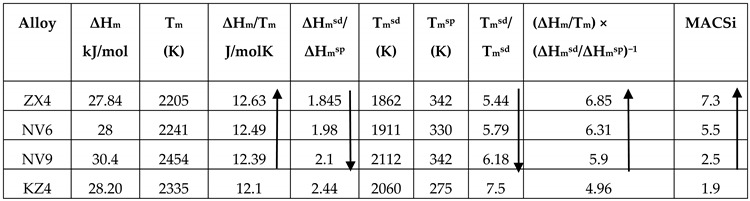
Alloy parameters for the macrosegregation of Si (MACSi) in cast alloys with Nb_3_Sn (alloys ZX4, NV6 [22] and NV9 [22]) and the alloy KZ4 [23] without Nb_3_Sn.

**Table 17:**
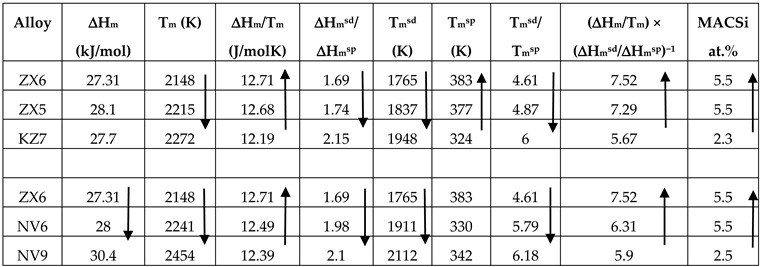
Alloy parameters for the macrosegregation of Si in in cast alloys with Nb_3_Sn (alloys ZX5 [13], NV6, NV9 [22], ZX6) and the alloy KZ4 [23] without Nb_3_Sn.

**Table 18:**
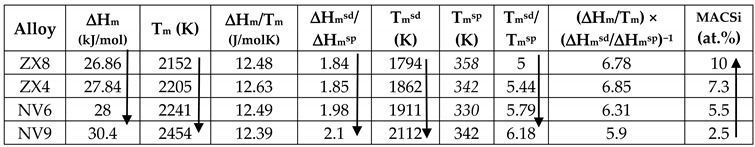
Alloy parameters for macrosegregation of Si in the Nb_3_Sn containing alloys ZX8, ZX4, NV6 [20], and NV9 [20].

**Table 19:** Comparison of the Si + Sn and Si + Sn + Al concentrations and the Si/Sn ratios in Nb_3_Sn in as cast and heat treated Nb-silicide based alloys with/out Ti and without Hf. Numbers rounded up to first decimal point.

Alloy *	Si + Sn	Si + Sn + Al	Si/Sn	Si + Sn	Si + Sn + Al	Si/Sn	Reference
	As cast			Heat treated			
ZX4	19.4		0.6	18.9		0.4	This work
ZX6	15.3	21.3	0.8	12.9	19.9	0.6	This work
ZX8	14.9	20.1	0.6	13.5	19.3	0.6	This work
NV6	18.2		0.7	16.3		0.5	20
EZ7	13.6	19.8	1.2	11.7	18	0.8	26
NV9	17.8		1	17.7		0.6	20

* NV6 = Nb-24Ti-18Si-5Sn, NV9 = Nb-18Si-5Sn, EZ7 = Nb-18Si-5Al-5Sn.

**Table 20:** Comparison of oxides in the scales, diffusion zones, and bulk microstructures in the oxidised alloys at 800 °C.

Alloy	Oxide Scale		Enrichment in Sn and Sn Rich Phase(s) Formation	Diffusion Zone		Bulk Microstructure
	Thickness (μm)	Oxides		Thickness (μm)	Phases	Phases
ZX4	10	Nb rich, Nb and Si rich, Ti rich ^+^	Yes	20	Nb_5_Si_3_, Nb_3_Sn, oxidized Nb_ss_	Nb_5_Si_3_, Nb_3_Sn, Nb_ss_
ZX6	10	Nb rich, Nb and Si rich	Yes	30	Nb_5_Si_3_, Nb_3_Sn, (Nb_5_Si_3_ + Nb_ss_) *	Nb_5_Si_3_, Nb_3_Sn, (Nb_5_Si_3_ + Nb_ss_)
ZX8	5	Nb rich, Nb and Si rich	Yes	10	Nb_5_Si_3_, Nb_3_Sn	Nb_5_Si_3_, Nb_ss_, Nb_3_Sn, Laves, (Nb_5_Si_3_ + Nb_ss_)

* oxidised structure. + no WDS data for this oxide type.

**Table 21:** Comparison of the Nb and Nb and Si rich oxides formed in the scales of the alloys at 800 °C.

Alloy	Nb + Ti	(Nb + Ti)/Si	Si + Sn	Si + Sn + Al	(Nb + Ti)/(Si + Sn)	(Nb + Ti)/(Si + Al + Sn)	Al + Cr + Sn	(Nb + Ti)/(Al + Cr + Sn)	Nb/Ti
**Nb rich oxide**									
ZX4	29.4	18.4	1.7		17.3		3.4	8.6	1.7
ZX6	28.3	21	1.9	4.2	14.9	6.8	2.8	10.1	1.6
ZX8	28.4	47.3	0.8	2.3	35.5	12.3	6.4	4.4	2.2
**Nb and Si rich oxide**									
ZX4	20	1.5	13.5		1.5		0.7	29.7	2.4
ZX6	22.7	2.4	9.9	11	2.3	2.1	1.6	14.1	2
ZX8	22.2	2.1	10.9	12.5	2	1.8	2.5	8.9	2.2

**Table 22:** Comparison of the compositions of the Nb_5_Si_3_, Nb_3_Sn, and Nb_ss_ in the bulk of the oxidised alloys at 800 °C. Note that for the solid solution there are only data for the alloy ZX4.

Alloy	Nb	Ti	Si	Cr	Al	Sn	O	Si + Sn	Si + Sn + Al	Nb/Ti
**Nb_5_Si_3_**										
ZX4	43.7	15.6	34	1		1.5	4.2	35.5		2.8
ZX6	42.9	16.5	33.7		2.5	2	2.4		38.2	2.6
ZX8	41	19.7	32.3	1.2	0.9	1	3.9		34.2	2.1
**Nb_3_Sn**										
ZX4	55	20.5	5.9	2.9		9.4	6.3	15.3		2.7
ZX6	53.4	22	5.1		4.6	8.6	6.3	13.7	18.3	2.4
ZX8	54.5	19.6	2.4	3.8	5.2	8.3	6.2	10.7	15.9	2.8
**Nb_ss_**										
ZX4	46.7	29.4	2.7	9.2		4.8	7.2	7.5		1.6

**Table 23:** Comparison of the alloys in terms of the scale thickness, oxides in the scale, thickness of Sn rich area, Sn rich intermetallics forming in the latter and phases in the bulk microstructure after oxidation at 1200 °C.

Alloy	Scale		Sn Rich Area		Bulk
	Thickness (μm)	Oxides	Thickness (μm)	Sn Rich intermetallics	Phases
ZX4	500	Nb rich *, Nb and Si rich *, Ti rich *	55	Nb_5_Si_3_, Nb_3_Sn, Nb_5_Sn_2_Si, NbSn_2_	Nb_5_Si_3_, Nb_ss_, Nb_3_Sn, Laves phase
ZX6	300	Nb rich, Nb and Si rich, Ti rich	50	Nb_5_Si_3_, Nb_3_Sn, Nb_5_Sn_2_Si	Nb_5_Si_3_, Nb_3_Sn
ZX8	250	Nb rich, Nb and Si rich, Ti rich	50	Nb_5_Si_3_, Nb_3_Sn, Nb_5_Sn_2_Si	Nb_5_Si_3_, Nb_3_Sn, Laves phase

* no WDS data for this oxide is available.

**Table 24:** Comparison of the Nb, Nb and Si, and Ti rich oxides formed in the scales of the high Sn alloys at 1200 °C. Note that there are no data for the alloy ZX4.

Alloy	Nb + Ti	(Nb + Ti)/Si	Si + Sn	Si + Sn + Al	(Nb + Ti)/(Si + Sn)	(Nb + Ti)/(Si + Al + Sn)	Al+Cr+Sn	(Nb + Ti)/(Al + Cr + Sn)	Nb/Ti
**Nb rich oxides**									
ZX6	32.1			1.3		24.7	1.3	24.7	3.1
ZX8	28.3		0.1	0.3	283	94.3	0.4	70.8	2.7
**Nb and Si rich oxides**									
ZX6	20.3	1.5	13.6	14	1.5	1.4	0.5	40.6	2.6
ZX8	19.8	2.3	8.8	9.3	2.3	2.1	1	19.8	2.7
**Ti rich oxides**									
ZX6	27.8	278	0.1	5.6	278	5	5.5	5	0.3
ZX8	24.7	124	0.6	2.5	41.2	9.9	5.8	4.3	0.4

**Table 25:** Comparison of the compositions of the Nb_5_Si_3_ Nb_3_Sn and Nb_ss_ in the bulk of the oxidised alloys at 1200 °C.

Alloy	Nb	Ti	Si	Cr	Al	Sn	O	Si+Sn	Si+Sn+Al	Nb/Ti
**Nb_5_Si_3_**										
ZX4	43.6	15.4	34.9	0.9		1.6	3.6	36.5		2.8
ZX6	43.1	15.6	33.9		1.5	1.4	4.5	35.3	36.8	2.8
ZX8	42	19	30.2	0.7	2.8	1.1	4.2	31.3	34.1	2.2
**Nb_3_Sn**										
ZX4	48.8	26.4	2.9	3.8		12.4	5.7	15.3		1.9
ZX6	54.2	23.5	3.1		5	8.6	5.6	11.7	16.7	2.3
ZX8	51.8	21.8	1.5	4.3	6.4	8.2	6	9.7	16.1	2.4
**Nb_ss_**										
ZX4	54.8	26.4	2.9	6.4		3.1	6.4	6	0.9	2.1

## Supplementary Materials

The following are available online at https://www.mdpi.com/1996-1944/13/1/245/s1. Table S1: Calibration standards and their compositions for the EPMA analyses, Table S2: Analysis data (at.%) of the as cast and heat treated alloy ZX4 (average values in bold numbers), Table S3: Analysis data (at.%) of the as cast and heat treated alloy ZX6 (average values in bold numbers), Table S4: Analysis data (at.%) of the as cast and heat treatment alloy ZX8 (average values in bold numbers).
