# Supplementary material for: A Study of the Effect of 5 at.% Sn on the Micro-Structure and Isothermal Oxidation at 800 and 1200 °C of Nb-24Ti-18Si Based Alloys with Al and/or Cr Additions
Source: Materials (Basel). 2020 Jan 6;13(1):245. doi: 10.3390/ma13010245 (PMC6981790; doi:10.3390/ma13010245)
Supplement: Supplementary file 1 [file materials-13-00245-s001.pdf]

materials

Supplementary Materials

A Study of the Effect of 5 at.% Sn on the Micro-Structure and Isothermal Oxidation at 800 and 1200 °C of Nb-24Ti-18Si Based Alloys with Al and/or Cr Additions

Zhen Xu, Claire Utton and Panos Tsakiropoulos *

Department of Materials Science and Engineering, Sir Robert Hadfield Building,
The University of Sheffield, Mappin Street, Sheffield S1 3JD, UK; zhen_xu@outlook.com (Z.X.), c.utton@sheffield.ac.uk (C.U.)
* Correspondence: p.tsakiropoulos@sheffield.ac.uk

Table S1. Calibration standards and their compositions for the EPMA analyses.

Elements	Reference Materials	Composition of the reference materials (wt.%)
Nb	Nb	Nb: 100%
Ti	Rutile	Ti: 59.34%, O: 39.89%, Mg: 0.01%, Fe: 0.59%, Nb: 0.17%
Si	Zircon	Si: 15.24%, Mn: 0.03%,Zr: 49.14%, Hf: 0.82%, O: 34.77%
Al	Spinel	Mg: 17.08%, Al: 37.93%, O: 44.99 %
Cr	Cr	Cr: 100%
Sn	Sn	Sn: 100%
O	Forsterite	O: 45.5%, Mg: 34.5%, Si: 20.0%

Table S2. Analysis data (at.%) of the as cast and heat treated alloy ZX4 (average values in bold numbers).

	Nb	Ti	Si	Cr	Sn
			As cast		
Surface*	46.1 ± 0.3	24.8 ± 0.3	19.5 ± 0.5	4.7 ± 0.1	4.9 ± 0.2
	45.6–46.5	24.3–25.3	18.8–20.4	4.4–4.9	4.8–5.3
Bulk*	46.5 ± 0.3	24.2 ± 0.9	20.3 ± 1.8	4.0 ± 1.1	5.0 ± 0.4
	45.8–46.7	22.2–25.3	17.8–22.0	3.5–4.9	4.2–5.6
Bottom*	44.7 ± 0.6	27.3 ± 1.0	16.7 ± 1.4	6.1 ± 0.7	5.2 ± 0.2
	44.1–45.7	25.7–28.8	14.7–19.1	5.2–7.1	4.9–5.6
Nb_{ss}	44.5 ± 2.6	35.0 ± 1.6	3.6 ± 0.8	11.3 ± 0.9	5.6 ± 0.5
	40.3–46.8	33.3–37.7	2.2–4.5	10.2–12.1	4.7–6.1
Nb_3Sn	55.8 ± 0.4	22.3 ± 0.5	7.5 ± 0.5	2.5 ± 0.3	11.9 ± 0.7
	55.5–56.4	21.6–23.0	6.8–8.4	2.2–3.0	10.7–12.5
Ti-rich	41.1 ± 0.4	22.1 ± 0.9	33.1 ± 1.4	1.9 ± 0.5	1.8 ± 0.2
Nb_5Si_3	40.7–41.7	20.9–23.4	31.1–34.3	1.1–2.7	1.4–2.1
Nb_5Si_3	45.2 ± 0.5	17.2 ± 0.4	34.8 ± 0.2	0.9 ± 0.1	1.9 ± 0.2
	44.5–45.8	16.7–17.9	34.5–35.2	0.8–1.1	1.7–2.2

Laves phase	20.8 ± 2.3<br>18.7–24.4	28.3 ± 4.2<br>21.0–34.7	10.7 ± 2.2<br>8.5–12.0	38.4 ± 3.9<br>34.0–44.8	1.8 ± 0.7<br>0.7–3.0
Heat treated					
Bulk*	44.6 ± 0.5<br>43.9–45.6	26.0 ± 0.5<br>25.0–26.4	20.0 ± 0.5<br>19.1–20.7	4.3 ± 0.2<br>4.1–4.6	5.1 ± 0.4<br>4.6–5.5
Nb_{ss}	52.1 ± 0.8<br>50.9–53.4	33.3 ± 0.8<br>32.3–34.2	0.7 ± 0.1<br>0.5–1.0	9.0 ± 0.4<br>8.7–9.7	4.9 ± 0.2<br>4.7–5.2
Nb_3Sn	49.8 ± 0.3<br>49.3–50.4	26.3 ± 0.3<br>25.8–26.8	5.2 ± 0.5<br>4.7–6.5	5.0 ± 0.2<br>4.6–5.2	13.7 ± 0.2<br>13.3–13.9
Nb_5Si_3	42.9 ± 0.9<br>41.7–44.4	19.5 ± 0.8<br>18.4–21.0	35.6 ± 0.6<br>34.8–36.2	0.5 ± 0.1<br>0.4–0.8	1.5 ± 0.4<br>1.1–2.2

* Large area analysis.

Table S3. Analysis data (at.%) of the as cast and heat treated alloy ZX6 (average values in bold numbers).

	**Nb**	**Ti**	**Si**	**Al**	**Sn**
As cast					
Top*	44.8 ± 0.1<br>44.6–45.1	26.3 ± 0.3<br>25.8–26.9	19.2 ± 0.3<br>18.8–19.7	4.8 ± 0.2<br>4.4–5.1	4.9 ± 0.2<br>4.7–5.2
Bulk*	45.1 ± 2.7<br>40.6–49.6	26.0 ± 1.7<br>23.3–29.4	18.9 ± 1.5<br>16.1–21.5	5.1 ± 0.3<br>4.7–5.6	4.9 ± 0.4<br>3.7–5.4
Bottom*	49.9 ± 0.3<br>49.4–50.3	23.2 ± 0.6<br>22.1–24.4	16.9 ± 0.5<br>16.0–18.0	4.9 ± 0.2<br>4.5–5.1	5.1 ± 0.1<br>5.0–5.4
Nb_{ss}	52.4 ± 3.6<br>45.9–56.9	34.1 ± 3.2<br>30.2–38.9	2.8 ± 0.7<br>1.8–4.3	5.9 ± 0.2<br>5.5–6.1	4.8 ± 0.3<br>4.5–5.4
Nb_5Si_3	45.7 ± 1.3<br>44.5–47.4	17.1 ± 1.4<br>15.3–18.3	32.5 ± 0.8<br>31.5–33.3	2.9 ± 0.5<br>2.2–3.5	1.8 ± 0.4<br>1.3–2.4
Ti rich<br>Nb_5Si_3	42.2 ± 1.2<br>40.1–43.5	20.9 ± 1.3<br>19.4–23.4	31.3 ± 0.3<br>30.6–31.7	4.0 ± 0.2<br>3.6–4.2	1.6 ± 0.2<br>1.2–1.8
Nb_3Sn	54.8 ± 1.4<br>52.7–57.2	24.0 ± 1.2<br>21.6–25.8	6.6 ± 0.4<br>6.1–7.3	5.9 ± 0.5<br>5.2–6.8	8.7 ± 1.0<br>7.3–9.9
Heat treated					
Bulk*	47.9 ± 0.2<br>47.6–48.2	23.9 ± 0.6<br>23.1–25.0	19.3 ± 0.9<br>17.7–21.2	4.5 ± 0.2<br>4.1–4.9	4.4 ± 0.2<br>4.0–4.7
Nb_5Si_3	46.5 ± 0.8<br>45.6–47.5	16.8 ± 0.7<br>15.8–17.6	33.2 ± 0.1<br>33.0–33.4	2.1 ± 0.1<br>2.0–2.4	1.4 ± 0.1<br>1.3–1.6

Ti-rich Nb_5Si_3	44.0 ± 0.8<br>43.2–45.6	19.2 ± 0.6<br>18.1–19.8	34.2 ± 0.4<br>33.5–34.6	1.7 ± 0.2<br>1.4–2.0	0.9 ± 0.2<br>0.7–1.2
Nb_3Sn	55.0 ± 0.5<br>53.9–55.5	25.1 ± 0.6<br>24.3–26.3	4.7 ± 0.3<br>4.2–5.0	7.0 ± 0.2<br>6.7–7.3	8.2 ± 0.1<br>8.0–8.4

* Large area analysis.

Table S4. Analysis data (at.%) of the as cast and heat treatment alloy ZX8 (average values in bold numbers).

**As Cast**	**Nb**	**Ti**	**Si**	**Cr**	**Al**	**Sn**
Surface*	41.3 ± 0.7<br>40.4–43.0	28.1 ± 1.1<br>25.8–30.0	14.1 ± 1.9<br>10.9–18.8	6.4 ± 0.7<br>4.8–8.0	5.2 ± 0.2<br>4.9–5.6	4.9 ± 0.3<br>4.3–5.3
Bulk*	45.8 ± 0.9<br>43.3–46.6	21.4 ± 0.9<br>20.3–23.9	19.7 ± 1.0<br>18.0–20.9	4.5 ± 0.8<br>3.3–5.3	4.3 ± 0.5<br>3.7–5.2	4.3 ± 0.2<br>4.0–4.8
Bottom*	45.4 ± 0.4<br>44.9–46.3	22.1 ± 0.3<br>21.6–22.6	17.7 ± 0.5<br>16.9–18.6	5.5 ± 0.3<br>5.0–6.2	4.8 ± 0.2<br>4.6–5.1	4.5 ± 0.1<br>4.3–4.8
Nb_5Si_3	42.0 ± 0.6<br>41.2–43.2	20.6 ± 0.5<br>19.9–21.4	31.9 ± 0.9<br>30.7–33.3	1.1 ± 0.2<br>0.8–1.4	2.6 ± 0.5<br>1.7–3.2	1.8 ± 0.4<br>1.2–2.2
Ti-rich Nb_5Si_3	35.2 ± 1.1<br>33.2–36.6	27.5 ± 1.2<br>26.2–29.9	29.2 ± 0.9<br>27.5–30.0	2.6 ± 0.6<br>1.9–3.6	3.7 ± 0.2<br>3.3–4.0	1.8 ± 0.2<br>1.6–2.3
Nb_3Sn	50.3 ± 1.3<br>48.0–52.0	26.2 ± 1.1<br>24.8–28.3	5.3 ± 0.2<br>5.0–5.6	3.4 ± 0.3<br>2.9–4.1	5.2 ± 0.4<br>4.6–6.2	9.6 ± 0.6<br>8.2–10.1
Laves phase	27.0 ± 1.6<br>25.9–28.8	20.8 ± 0.6<br>20.1–21.3	9.6 ± 1.8<br>8.4–11.7	38.8 ± 2.5<br>36.7–41.6	2.7 ± 0.4<br>2.3–3.1	1.1 ± 0.4<br>0.7–1.6
Heat treated	Nb	Ti	Si	Cr	Al	Sn
Bulk*	46.6 ± 0.7<br>45.9–47.5	21.1 ± 0.8<br>20.3–22.2	18.6 ± 1.1<br>17.4–20.4	5.1 ± 0.4<br>4.6–5.7	4.0 ± 0.2<br>3.8–4.5	4.6 ± 0.3<br>4.0–5.0
Nb_5Si_3	45.3 ± 0.6<br>44.7–46.1	17.1 ± 0.7<br>16.1–17.7	34.3 ± 0.2<br>34.1–34.5	0.7 ± 0.1<br>0.6–0.9	1.5 ± 0.2<br>1.3–1.7	1.1 ± 0.2<br>0.9–1.2
Ti rich Nb_5Si_3	34.7 ± 0.6<br>33.9–35.4	27.0 ± 0.9<br>26.2–28.4	32.5 ± 0.6<br>31.8–33.3	2.3 ± 0.2<br>2.0–2.6	3.2 ± 0.1<br>3.0–3.3	0.3 ± 0.1<br>0.3–0.4
Nb_3Sn	51.9 ± 0.4<br>51.4–52.5	22.5 ± 0.6<br>21.6–23.4	4.8 ± 0.2<br>4.2–5.0	6.3 ± 0.6<br>5.8–7.6	5.8 ± 0.2<br>5.4–6.1	8.7 ± 0.1<br>8.5–8.9
Nb_{ss}	50.4 ± 2.7<br>48.1–54.6	32.0 ± 2.4<br>28.5–34.4	0.6 ± 0.3<br>0.4–1.1	9.2 ± 0.5<br>8.4–9.6	6.0 ± 0.5<br>5.1–6.5	1.8 ± 0.2<br>1.5–2.0
Laves phase	27.3 ± 0.4<br>26.6–27.8	13.7 ± 3.6<br>11.2–20.0	8.3 ± 1.89<br>6.1–10.4	48.5 ± 2.2<br>45.1–50.6	1.9 ± 0.2<br>1.6–2.1	0.3 ± 0.2<br>0.1–0.6

* Large area analysis.
